# Next-generation diabetes diagnostics: spectrochemical staging of red blood cells using vibrational spectroscopy

**DOI:** 10.1016/j.jare.2026.01.052

**Published:** 2026-01-19

**Authors:** Aleksandra Wilk, Natalia Wilkosz, Marzena Rugiel, Marta Saluga, Kamil Kawon, Tetiana Stepanenko, Katarzyna Bulat, Aleksandra Wajda, Michał Szuwarzynski, Anna Zajaczkowska, Anna Morgunowicz, Grzegorz Chodaczek, Víctor Navarro-Esteve, David Perez-Guaita, Joanna Chwiej, Katarzyna M. Marzec

**Affiliations:** a AGH University of Krakow, Faculty of Physics and Applied Computer Science, al. A. Mickiewicza 30, 30-059 Krakow, Poland; b Jagiellonian University, Doctoral School of Exact and Natural Sciences, Lojasiewicza 11, 30-348 Krakow, Poland; c Jagiellonian University, National Synchrotron Radiation Centre SOLARIS, Czerwone Maki 98 Str., 30-392 Krakow, Poland; d Jagiellonian University, Faculty of Chemistry, Gronostajowa 2, 30-387 Krakow, Poland; e Faculty of Pharmacy, Jagiellonian University Medical College, 9 Medyczna Street, Krakow 30-688, Poland; f Academic Centre for Materials and Nanotechnology, AGH University of Krakow, Al. Mickiewicza 30, 30-059 Krakow, Poland; g Lukasiewicz Research Network - PORT Polish Center for Technology Development, Stabłowicka 147, 54-066 Wrocław, Poland; h Department of Analytical Chemistry, University of Valencia, Dr. Moliner 50, Burjassot, Spain; i EBEAM Centre, Nanotechnology Centre, CEET, VSB–Technical University of Ostrava, Ostrava 708 00, Czech Republic

**Keywords:** Red blood cells (RBCs), Type 2 Diabetes Mellitus (T2DM), Vibrational Spectroscopy, Fourier Transform Infrared Spectroscopy (FT-IR), Raman Spectroscopy (RS), Spectrochemical Markers

## Abstract

**Introduction:**

Type 2 diabetes mellitus (T2DM) induces progressive biochemical and morphological alterations in red blood cells (RBCs), driven by hyperglycemia-induced oxidative stress and disrupted lipid–protein homeostasis. These changes compromise membrane integrity and serve as sensitive molecular indicators of disease severity. Yet, a non-invasive strategy for capturing such alterations at the molecular level remains an unresolved challenge.

**Objectives:**

This study employs Fourier Transform Infrared Spectroscopy (FT-IR) and Raman Spectroscopy (RS) to uncover sex- and age-specific molecular alterations in RBCs induced by T2DM. By integrating classical and advanced analytical techniques, we identify novel spectrochemical markers of disease progression, offering a non-invasive, label-free framework for staging and monitoring diabetic pathology at the molecular level.

**Methods:**

A total of 120 db/db and C57BL/6J mice were tested at 7, 12 and 24 weeks of age. Standard hematological and biochemical analyses were combined with vibrational spectroscopy (FT-IR, RS) and atomic force microscopy (AFM), to assess RBC morphology and molecular composition. Spectral data was analyzed using univariate and multivariate statistics to identify disease-specific spectral markers.

**Results:**

Partial Least Squares Discriminant Analysis reliably distinguished diabetic and control RBC profiles, confirming the presence of robust, disease-specific molecular patterns. Integration with hematological and biochemical parameters further validated the diagnostic relevance of this label-free, non-invasive approach. Structural protein analysis revealed a consistent decline in α-helical content and an increase in β-sheet and β-turn structures, reflecting protein misfolding and aggregation, particularly in older diabetic females. Alterations in disulfide bonding, hydrated β-sheets and H-bonded antiparallel β-sheets highlighted oxidative stress-mediated membrane destabilization. Additionally, elevated plasma triglyceride levels and increased saturation of RBC membrane lipids indicate impaired lipid handling.

**Conclusion:**

Vibrational spectroscopy enables non-invasive, molecular-level profiling of diabetes-induced RBC alterations, revealing sex- and age-dependent signatures of disease progression. This approach offers a promising platform for precise staging and stratification in metabolic disorders.

## Highlights

• Spectral biomarkers show age- and sex-specific diabetes progression in RBCs.
• FT-IR and Raman detect protein misfolding and membrane lipid remodeling in T2DM.
• Disease-specific spectral profiles correlate with metabolic and hematologic changes.
• Study includes 120 mice with robust statistical analysis across three timepoints.
• Label-free, non-destructive method supports translational use in metabolic disorders.

## Graphical abstract

**Figure:**
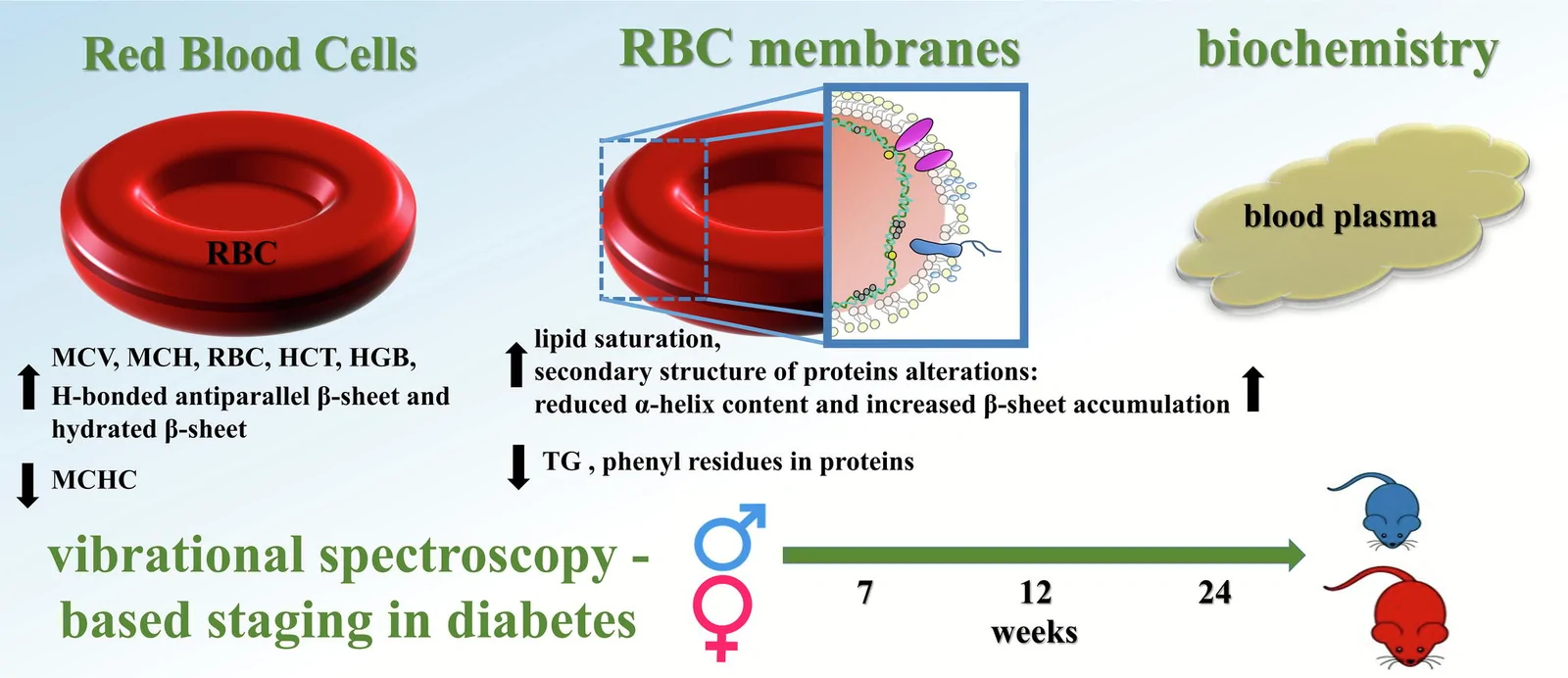

## Introduction

Type 2 diabetes mellitus (T2DM) is a rapidly growing global health challenge, affecting nearly 500 million people worldwide, according to the WHO (2021) [1,2]. Despite advancements in understanding diabetes pathogenesis, its molecular and cellular effects on red blood cells (RBCs) remain underexplored. T2DM is associated with protein misfolding and aggregation, a phenomenon well-documented in neurodegenerative disorders such as Alzheimer’s and Parkinson’s disease but less studied in diabetes [3]. Most research in this area has focused on pancreatic β-cell dysfunction, particularly the accumulation of islet amyloid polypeptide (IAPP) aggregates, a hallmark of T2DM [4,5]. However, diabetes also induces significant structural and biochemical changes in RBCs, leading to alterations in membrane composition, mechanical properties and protein stability [6,7]. These changes contribute to vascular dysfunction, oxidative stress and increased hemoglobin glycation, all of which play a crucial role in diabetic complications such as microangiopathy [8,9].

RBCs rely on a delicate balance of membrane proteins and lipids to maintain their structural integrity, deformability and oxygen transport capacity [10,11]. Protein misfolding and aggregation in RBC membranes, along with lipid alterations, may serve as early-stage biomarkers for diabetes progression and complications. Disruptions in secondary and tertiary protein structures can cause increased membrane rigidity, impaired RBC turnover and enhanced oxidative damage [12,13]. Understanding these molecular changes in RBCs may offer new diagnostic insights into T2DM pathophysiology and potential targets for therapeutic intervention.

Given the progressive nature of diabetes, defining molecular biomarkers that differentiate early and advanced disease stages is crucial for diagnostics and monitoring. While hematological and lipidomic assessments provide valuable insights, non-invasive molecular characterization of RBCs remains limited. Advanced vibrational spectroscopy techniques—Fourier Transform Infrared Spectroscopy (FT-IR) and Raman Spectroscopy (RS)—offer a powerful approach for investigating diabetes-related structural modifications in RBC membranes and cytoplasmic proteins. These methods provide high-resolution molecular signatures, allowing for the identification of early-stage disease markers and tracking protein misfolding, lipid remodeling and oxidative stress responses [12,13]. Previous research has demonstrated the utility of vibrational spectroscopy for biochemical fingerprinting of RBCs, including disease-specific protein and lipid alterations [[14], [15], [16], [17]].

FT-IR spectroscopy effectively distinguishes various protein conformations, such as α-helix, β-sheet and β-turn, as well as has been widely applied in biomedical research to track protein misfolding and aggregation [[18], [19], [20], [21]]. The technique has been successfully utilized for protein secondary structure analysis in diverse conditions, including Alzheimer’s disease, HIV treatments and autoimmune disorders [[22], [23], [24]]. Additionally, recent advancements in FT-IR methodology have enhanced its precision, further solidifying its role as a powerful tool for biomolecular analysis [[25], [26], [27]].

Complementarily, Raman spectroscopy (RS) provides detailed insights into peptide bond arrangements and protein structural integrity, making it an invaluable tool for studying conformational changes in disease states [28]. In FT-IR, secondary structure analysis is primarily based on Amide I and Amide II bands, providing information about protein folding states [29,30]. The second-derivative analysis of these bands enables the identification of β-sheet, β-turn and α-helix structures, revealing protein misfolding patterns associated with diabetes progression [31,32]. Additionally, disulfide bond content, crucial for protein stability and oxidative stress response, can be detected through both FT-IR and RS, providing further insights into diabetes-induced RBC damage [33,34]. Furthermore, the wealth of information contained in the IR and RS spectrum is not always represented in single bands, and therefore the machine learning algorithms are used to extract spectral markers associated with a specific condition [[35], [36], [37]]. These methodologies can complement the use of quantitative single band integration providing information about shifts in the bands of interest as well as relative changes in intensities in the ratios of bands. For example, orthogonal Partial Least Squares Discriminant Analysis (oPLS-DA) enables the creation of discriminant models that not only highlight the importance of each spectral variable but also reveal the direction of their association with the studied classes studied [38]. Thus, the study of internal model features such as variable important projection (VIP) score as well as the component loading vector play a key role on the discovery of spectral markers.

To investigate diabetes-induced RBC alterations, we employed the widely used db/db mouse model, which recapitulates many key features of human T2DM pathophysiology [39]. This model is characterized by a mutation in the leptin receptor gene, leading to defective leptin signalling and a cluster of metabolic abnormalities that phenocopy several hallmarks of human T2DM [39]. Although the db/db model has been extensively studied, the impact of age and sex on RBC protein structure and function, as well as on membrane lipid organization, remains largely unexplored. However, as with all experimental models, db/db mice cannot fully reproduce the complexity of human T2DM. Species-specific differences in erythrocyte metabolism, membrane lipid composition and systemic lipid handling need to be taken into account when interpreting our findings and limit the direct extrapolation of murine RBC phenotypes to human clinical disease.

In this study, we systematically analyzed both male and female *db/db* mice and age-matched controls (C57BL/6J − hereafter referred to as C57 mice) at 7, 12, and 24 weeks, enabling us to differentiate diabetes progression stages. By integrating hematological, biochemical, spectroscopic analyzes as well as univariate and multivariate statistics, we aimed to define spectrochemical markers of diabetes-related RBC dysfunction and establish a molecular framework for disease staging.

By integrating FT-IR and RS for RBC analysis, we identified statistically significant structural alterations in RBC membrane proteins, offering a novel spectrochemical approach for monitoring diabetes-related erythrocyte dysfunction. Although vibrational spectroscopy has been used to probe diabetes-related alterations in RBCs [[40], [41], [42], [43]], previous studies have largely been restricted to single time points or unstratified cohorts. Here, we advance the field by providing, the first multimodal, sex-resolved and age-resolved spectrochemical analysis of RBC remodelling in the db/db model, enabled by a substantially expanded cohort. This integrative framework uncovers progressive and sex-specific biochemical signatures that were not accessible to earlier approaches. A comprehensive analysis of RBC isolated from *db/db* mice has not only enhanced our understanding of the relation between RBC pathology and the diabetes progression in males and females but also may facilitate the development of targeted sex-dependent therapeutic strategies to manage dyslipidemia in T2DM. Furthermore, this study demonstrates the power of vibrational spectroscopy in analyzing RBC membrane protein structures, reinforcing their potential as next-generation diagnostic tools for T2DM. By establishing a systematic spectrochemical staging framework, this research provides new avenues for early diabetes detection, personalized disease monitoring and biomarker discovery.

## Materials and methods

### Animal models and blood collection

DB/J (BKS.Cg-*Dock7^m^* +/+ *Lepr^db^*/J, *db/db)* mice and their wild-type controls, C57BL/6J (C57) were derived from Charles River (Italy) and housed in the Animal Facility of the Łukasiewicz Research Network − PORT Polish Center for Technology Development at a density of 3 to 4 mice per cage, housed in a temperature- and humidity-regulated environment with a 12-hour light/dark cycle, with continuous access to drinking water and standard rodent chow. The weight of animals was regularly monitored on a vivarium’s laboratory scale. For the experiments, healthy control mice (C57, N = 9–12) and diabetic mice (*db/db*, N = 9–12) were utilized at 7, 12, and 24 weeks of age (N = 120 in total). Whole blood samples were obtained with heparin (10 units/μl) as an anticoagulant. Mice were euthanized by cervical dislocation. The process was confirmed by total blood collection from the heart right ventricle through rib cage incision. White blood cells (WBC), red blood cells (RBC), hemoglobin (HGB), hematocrit (HCT), mean corpuscular volume (MCV), mean corpuscular hemoglobin (MCH), and mean corpuscular hemoglobin concentration (MCHC) were evaluated using a hematology analyzer (Scil Vet abc Plus, Horiba Medical, France). Hematologic data were examined with the use of OriginPro software (version 9.7.5.184).

All experiments were performed according to the Polish legislation, the ACT of January 15, 2015, on the protection of animals used for scientific or educational purposes and to the Directive 2010/63/EU (Chapter I, Article 3, Clause 1) of the European Parliament and of the Council. According to the latter, no additional approval is necessary when the animals sacrificed for tissue and blood collection are not subjected to any procedures beforehand. In this work, animals were not subject to any experimental procedures and only animal organs and tissues were used for analyses. In agreement with the Local Ethics Committee at the Institute of Immunology and Experimental Therapy, Polish Academy of Sciences in Wroclaw, an ethical review by was not required. All animal work was conducted in compliance with the ARRIVE guidelines.

### Isolation of RBCs, membrane separation processes and analysis of biochemical compounds

Whole blood was subjected to gentle centrifugation (acceleration: 800 × g, 10 min, 21 °C, soft braking) within 1 h of collection. After centrifugation, the supernatant was removed along with the remaining buffy coat, which contains white blood cells and platelets. Plasma was used for biochemical analysis of glucose, total cholesterol (TC), high-density lipoprotein (HDL), low-density lipoprotein (LDL), triglyceride (TG), phosphorus (P), creatinine (CR) and lactate dehydrogenase (LDH) levels using a biochemical analyzer (AU680/AU5800, Beckman Coulter, Brea, USA). Insulin was assessed via ELISA (Ultra-Sensitive Mouse Insulin and Mouse Hemoglobin A1c, Crystal Chem, Elk Grove Village, USA) using the manufacturer’s protocol. The RBCs isolation procedures were based on our earlier work [40] with one modification: the buffer was changed to a PBS buffer solution. To ensure consistent stabilization of samples across AFM and spectroscopic modalities, RBCs and RBC membranes were fixed using 0.5 % glutaraldehyde. This concentration was selected based on our previous systematic evaluation [44] of fixation effects across a 0–2 % range using ATR-FTIR, transmission FT-IR, Raman (488/1064 nm) and nano-FTIR in both RBC from healthy and diseased mouse models. That study demonstrated that 0.5 % glutaraldehyde preserves cell morphology while avoiding the biochemical artefacts observed at higher fixative concentrations, particularly in oxidatively stressed membranes. Thus, 0.5 % fixation provides an optimal balance between morphological stabilization and preservation of native biochemical signatures, enabling reproducible multitechnique analysis across the entire N = 120 cohort.

### FT-IR spectroscopy with data analysis and interpretation

All RBC membrane samples were examined using FT-IR measurements. For this purpose, the samples were placed on CaF_2_ slides (Crystran, USA). The measurements were performed in transmission mode with a Nicolet iN10 MX Infrared Microscope (Thermo Fisher Scientific, USA). FT-IR measurements and data analysis were performed according to procedures presented in detail in our previous works [[44], [45], [46]]. Supplemental Fig. S1 present the averaged IR spectra RBC membranes C57BL/6J (solid line) and *db/db* (dotted line) mice of both sexes (Male/M: Blue, Female/F: Red) at 7, 12, and 24 weeks of age. Supplemental Fig. S2 presents the most important regions taken into analysis.

### RS data collection, analysis and processing

Raman measurements were conducted with a confocal WITec Alpha300R Raman microscope (WITec GmbH, Ulm, Germany) equipped with an air-cooled solid-state laser operating at wavelengths of 532 and 488 nm (detailed in Supplementary Materials Figs. S3-S4), the UHTS 300 spectrometer (600 gratings/mm). A Zeiss EC Epiplan-Neofluar air objective (100×/0.90NA) was utilized, with the laser power at the sample position approximately 10 mW for the 532 nm laser and 5 mW for the 488 nm laser. The spectrum integration time was set to 3 s, with 10 accumulations taken per spectrum, yielding a spectral resolution of 3 cm^−1^ Raman measurements and initial preprocessing (removal of cosmic rays and background subtraction) were executed using WITec software (WITec Project Plus 5.3). All acquired Raman spectra were averaged from 90 single measurements. Further processing of the Raman spectra was carried out using OriginPro software (version 9.7.5.184), where integral intensities for selected Raman bands were calculated by delineating the area under each band and proper ratios were calculated. Supplemental Figs. S3-S4 present the averaged RS spectra RBC membranes C57BL/6J (solid line) and *db/db* (dotted line) mice of both sexes (Male/M: Blue, Female/F: Red) at 7, 12, and 24 weeks of age across the 400–3200 cm^−1^ range obtained with 532 and 488 nm excitations, respectively. Supplemental Figs. S5-S6 present the most important regions taken into analysis for RS with 532 nm and 488 nm excitations, respectively.

### AFM data acquisition and analysis

For AFM studies, isolated RBC samples fixed with 0.5 % glutaraldehyde were prepared as smears on CaF_2_ windows. These thin films of RBCs were air-dried for approximately 15 min. AFM images were collected exclusively for monolayers of RBCs. AFM measurements were performed using a Dimension Icon XR atomic force microscope (Bruker, Santa Barbara, CA, USA), operating in air in Peak Force Tapping (PFT) mode. Measurements utilized conventional silicon cantilevers (Scanasyst-AIR, Bruker) with a nominal spring constant of 0.4 N/m and a tip radius of less than 10 nm. AFM image collection and data analysis were performed according to procedures presented in detail in our previous works [44,47,48].

### Statistical and multivariate analyzes

The hematological, biochemical, IR, RS and AFM data were analyzed using OriginPro software, Version 2024 (OriginLab Corporation, Northampton, MA, USA). The normality of the data distribution was evaluated with the Shapiro-Wilk test. Depending on the distribution of the variables, comparisons were made using either parametric ANOVA (with Tukey’s post hoc test) or non-parametric Mann-Whitney tests: #*P* < 0.05, *P < 0.01, **P < 0.001, ***P < 0.0001. Specifically, the Mann-Whitney test was applied for comparisons of glucose and insulin levels, body weight and hematological and biochemical data, while one-way ANOVA was applied to assess changes in the height-to-diameter ratio of RBCs (based on AFM topography) and the ratios of integral intensities for selected IR and RS bands. Two statistical approaches were employed to summarize central tendencies and data distribution: (1) medians with interquartile ranges and min–max whiskers and (2) means with standard deviation whiskers. Pairwise comparisons were conducted to assess statistical variations between sexes of *db/db* mice and C57 controls over the study period (7 to 24 weeks). Hematological and biochemical blood parameters are additionally summarized in Supplemental Table 1, which includes mean values, standard deviations and the number of specific measurements. Data were presented in two formats: (1) boxplots displaying the median, interquartile range and minimum-to maximum values, and (2) interval plots depicting mean values with standard deviation whiskers. The Shapiro-Wilk test was used to assess normality, and statistical significance was evaluated using the Mann-Whitney test. The analyzed parameters were studied between 7 and 24 weeks of age in both *db/db* and control C57 mice.

Multivariate vibrational analysis was carried out in MATLAB 2024b (MathWorks Inc., Natic, USA) using in-house written scripts and machine learning toolbox, the PLS Toolbox (Eigenvector Research Inc., Manson, USA). FT-IR spectra underwent Savitzky–Golay second derivative (2nd order polynomial, 11 smoothing points), standard normal variate (SNV) scaling, mean centering and the 3100–2800 and 1800–900 cm^−1^ regions were selected for the RBC membrane FT-IR oPLS-DA. Raman spectra underwent second derivative and mean centering and the 3100–2800 and 1800–400 cm^−1^ regions were selected for its corresponding oPLS-DA. These models were created and optimized with the calibration dataset using the leave-one-animal-out (LOAO) cross-validation (CV) to select the optimal number of latent variables and their statistical significance was estimated by permutation testing. The permutation test involved the permutation of the vector of control/diabetic class while ensuring that all samples obtained from the same mice are associated with the same tag. Subsequently, an oPLS-DA model was developed using the modified dataset and the LOAO CV error of classification of the permuted model was estimated. This step was iterated with all possible combinations, obtaining a null distribution of classification errors that was used to compute the p-value for the original unpermuted dataset as the fraction of permuted models with lower classification error than in the unpermuted oPLS-DA. For FT-IR whole RBCs analysis of the 3100–2800 and 1800–900 cm^−1^ regions, support vector machine discriminatory analysis (SVM-DA) was used after PLS compression. The radial basis function (RBF) was chosen as the kernel function. The optimal parameters, (C and γ), were determined using a grid search approach, aiming to minimize CV error. The range explored for C and γ values were 10^−3^ to 100 and 10^−6^ to 10, respectively. Both LOAO CV and a 10 splits Venetian Blinds CV (i.e., 10-fold CV with systematic interleaving) were explored as CV methods.

## Results and Discussion

### Morphological and biochemical characteristics of the *db/db* mouse model of type 2 diabetes

We examined and compared body weight, glucose and insulin levels (Fig. 2) as well as blood morphological parameters (Fig. 3 and Supplemental Fig. S7A, A’), between *db/db* mice and non-diabetic C57 controls at 7, 12 and 24 weeks of age. By the age of 7 weeks, *db/db* mice displayed significantly elevated glucose levels, with averages of approximately 27.6 ± 4.3 mmol/l in males and 23.1 ± 4.5 mmol/l in females (Fig. 2A). Hyperglycemia increased rapidly, reaching 37.5 ± 5.2 mmol/l in males and 25.9 ± 7.4 mmol/l in females by 12 weeks. At 24 weeks, *db/db* mice displayed severe hyperglycemia, with glucose levels of 32.9 ± 3.1 mmol/l in males and 28.7 ± 7.6 mmol/l in females.

**Fig. 1:**
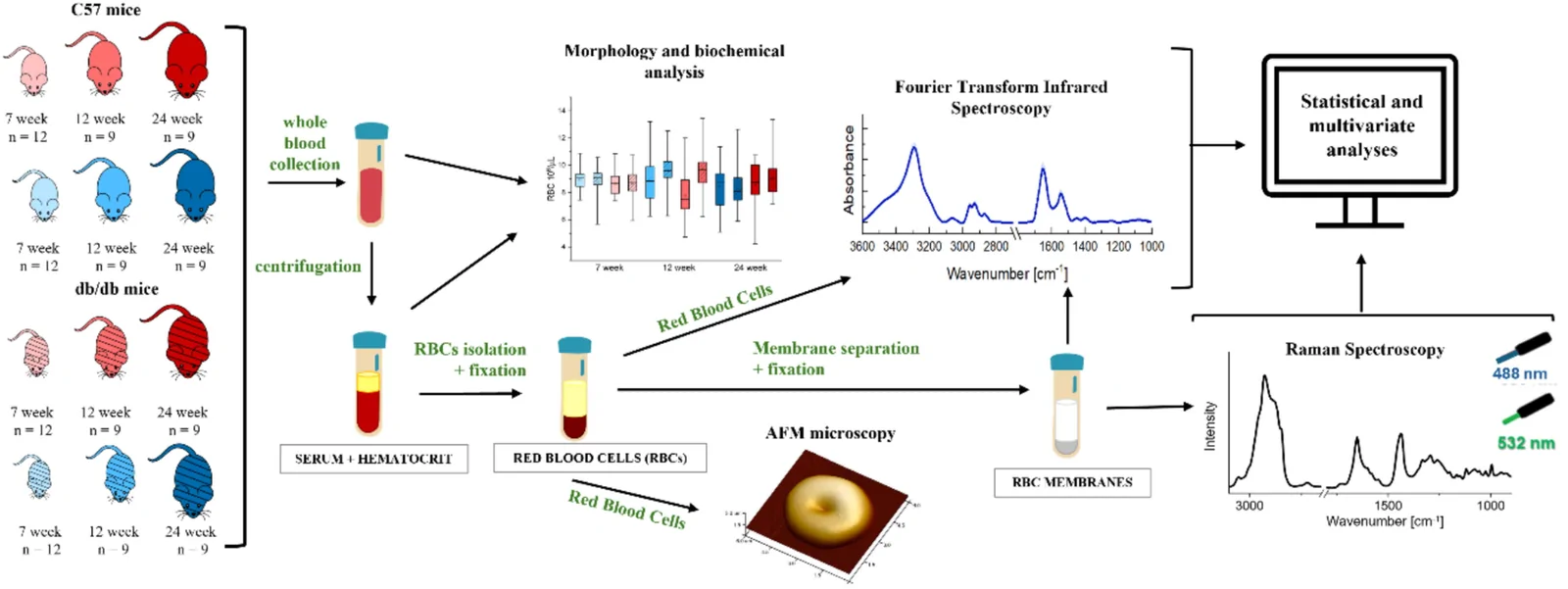
Schematic representation summarizing the measurement concepts across all applied techniques.

**Fig. 2:**
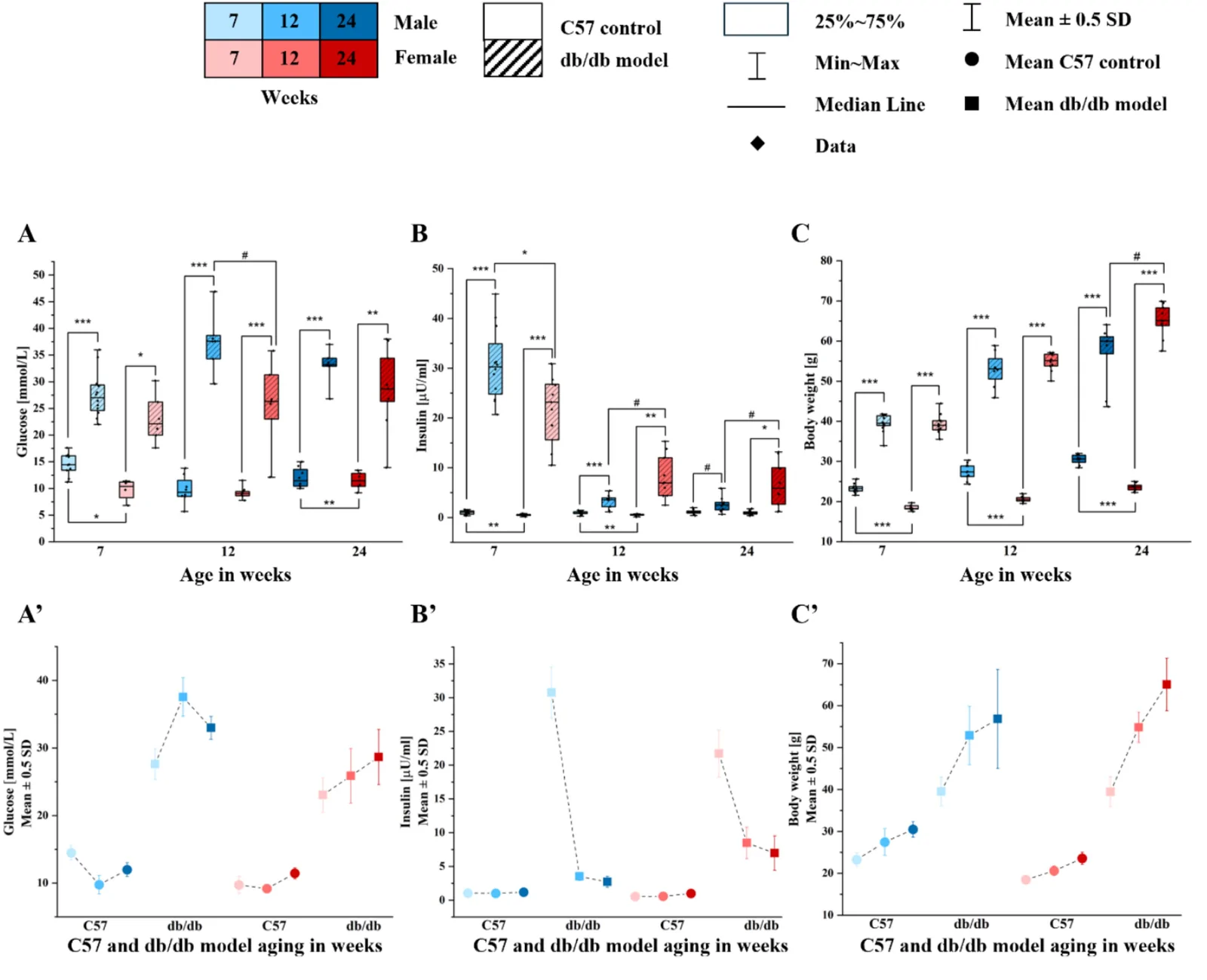
Changes in (A) glucose concentration [mmol/L], (B) insulin concentration [μU/mL] and (C) body weight [g] during the progression of diabetes (at 7, 12, and 24 weeks of age) in male and female db/db mice compared to C57 control mice are illustrated. The data (A-C) are presented as box plots displaying the median**,** interquartile range, and min–max whiskers. The Shapiro-Wilk test was used to assess normality. Statistical significance was determined using the Mann-Whitney test (#P < 0.05, *P < 0.01, **P < 0.001, ***P < 0.0001). Changes in the mean values of glucose and insulin concentration as well as body weight (A’-C’) over the course of diabetes progression (at 7, 12, and 24 weeks of age) in male and female db/db mice, compared to age-matched C57 controls, are also depicted. The data distribution (A’-C’) is presented as interval plots, where the mean values are shown with standard deviation whiskers. A dashed line connects the mean values to indicate the general trend in the analyzed parameters from 7 to 24 weeks of age in both db/db and control C57 mice.

**Fig. 3:**
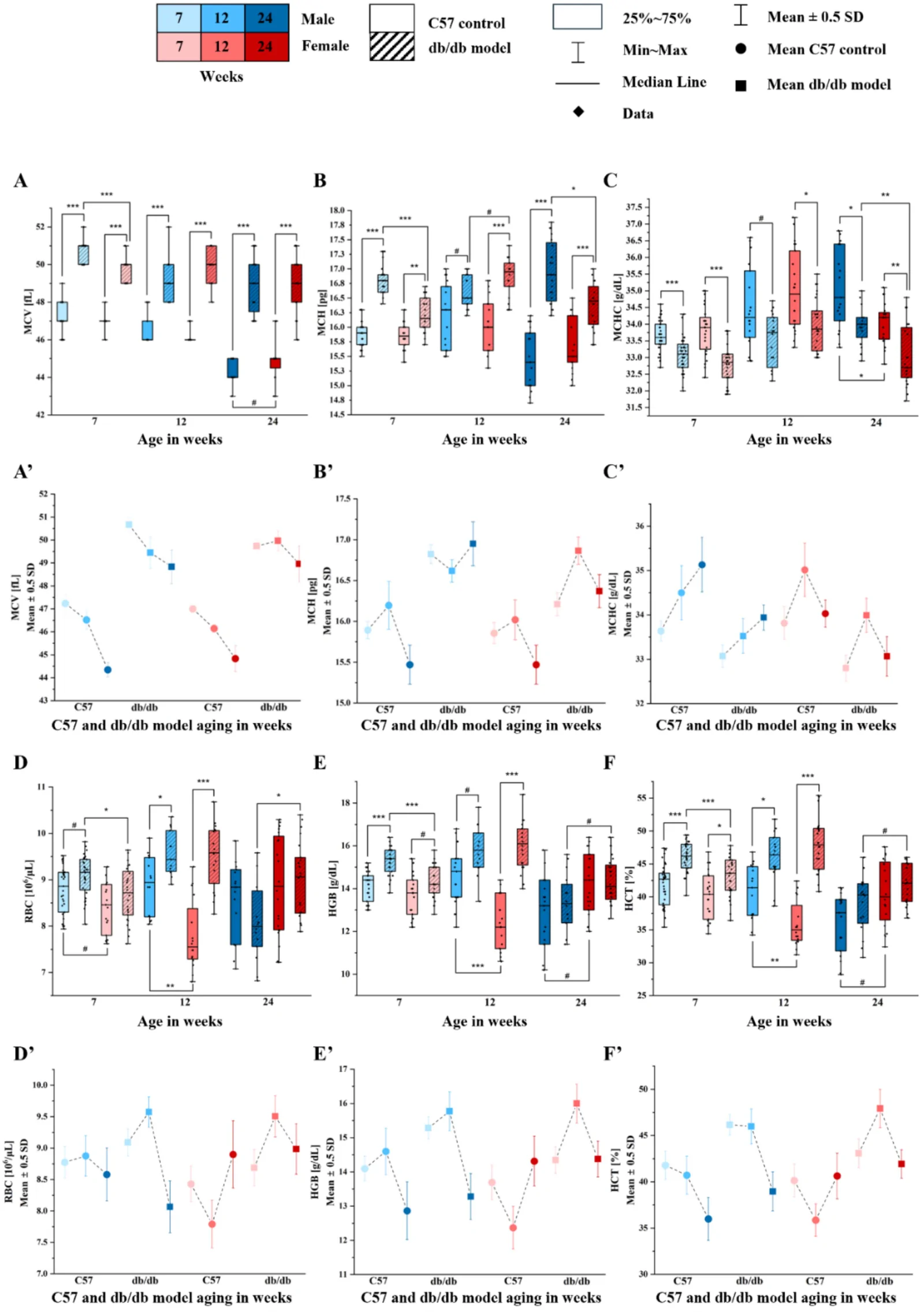
Changes in blood count (A-F) during the progression of diabetes (at 7, 12, and 24 weeks of age) in male and female db/db mice compared to C57 control mice are illustrated. The data (A-F) are presented as box plots displaying the median**,** interquartile range, and min–max whiskers. The Shapiro-Wilk test was used to assess normality. Statistical significance was determined using the Mann-Whitney test (#P < 0.05, *P < 0.01, **P < 0.001, ***P < 0.0001). Changes in the mean values of blood parameters (A’-F’) over the course of diabetes progression (at 7, 12, and 24 weeks of age) in male and female db/db mice, compared to age-matched C57 controls, are also depicted. The data distribution (A’-F’) is presented as interval plots, where the mean values are shown with standard deviation whiskers. A dashed line connects the mean values to indicate the general trend in the analyzed parameters from 7 to 24 weeks of age in both db/db and control C57 mice. Abbreviations: RBC – Red Blood Cells, HGB – Hemoglobin, HCT – Hematocrit, MCV – Mean Corpuscular Volume, MCH – Mean Corpuscular Hemoglobin, MCHC – Mean Corpuscular Hemoglobin Concentration. (For interpretation of the references to colour in this figure legend, the reader is referred to the web version of this article.)

In contrast, glucose concentration in C57 mice, the non-diabetic reference group, remained within the normal range, with no gender-dependent differences. This clearly indicates that already in early stages, *db/db* mice show diabetes-related symptoms, marked by substantial hyperglycemia, while control mice maintain normal glucose levels. At 7 weeks, *db/db* mice had insulin levels 30 to 40 times higher than those of control mice. However, a sharp decrease was observed in *db/db* mice from 30.7 ± 7.3 µU/ml to 3.5 ± 1.1 µU/ml by 12 weeks of age. Conversely, C57 mice maintained stable, low plasma insulin concentrations, ranging from 0.55 ± 0.16 pmol/l to 1.1 ± 0.4 pmol/l throughout the experiment. These data suggest that *db/db* mice initially show significantly elevated insulin levels compared to control mice, which remain stable throughout their lifespan. Male *db/db* mice were 169 % and females 204 % of the weight of non-diabetic mice, by 7 weeks of age (Fig. 2C). They gained weight rapidly, and by 12 weeks, males were 193 %, and females 266 % of the weight of the control group. By 24 weeks, *db/db* mice weighed approximately 286 % of control mice, with females weighing more than males. This rapid and extreme weight gain in *db/db* mice highlights their early-onset obesity, a key characteristic of this T2DM model. Starting at 7 weeks of age, *db/db* mice develop pronounced obesity and significant hyperglycemia in both blood and urine, which worsens with age. Despite hyperinsulinemia, indicating insulin presence, these mice develop insulin resistance, showing that insulin is no longer effective in regulating blood glucose. Moreover, around 12 weeks, plasma insulin levels decline, signalling β-cell failure [49].

The comparison between the examined mouse models of T2DM and non-diabetic controls, focusing on the RBC count, HGB, HCT, MCV, MCH, MCHC is shown in Fig. 3. The WBC count is shown in Supplemental Fig. S7. As shown in Fig. 3, the changes observed in blood indices, such as RBC, HGB, HCT, MCV, MCH, and MCHC, are significantly linked to diabetic pathology.

Significant differences in MCV, MCH, and MCHC were observed between non-diabetic C57 controls and *db/db* mice, consistent with previously reported alterations in erythrocyte rheology in diabetic models. Throughout the study (7, 12, and 24 weeks), MCV and MCH increased in diabetic mice of both sexes, whereas MCHC significantly decreased compared to controls (Fig. 3A–C).

Our findings align with a previous study [50], which reported increased MCV and MCH in diabetic female C57 BLKS/J-m +/+ Lpr db mice, though MCHC was not assessed. This supports the notion that larger erythrocytes circulate in diabetic mice, as previously suggested. Over 24 weeks, MCV declined in both groups, except for a transient, no statistically significant increase (P *>* 0.05) in diabetic females between 7 and 12 weeks, suggesting distinct aging patterns in males and females (Fig. 3A’). Our previous work [44,51] similarly demonstrated higher MCV in atherosclerotic mice versus controls, with age-related decline. Sex-specific MCH differences emerged only in diabetic mice (Fig. 3B’), where diabetic males exhibited a unique trend a decrease from 7 to 12 weeks, followed by an increase from 12 to 24 weeks unlike other groups. The most pronounced sex-specific effects were in MCHC (Fig. 3C’): values increased steadily in males, while in females, they rose from 7 to 12 weeks before declining by week 24. However, no statistically significant differences (P *>* 0.05) in MCHC were found between healthy and diabetic individuals of the same sex.

Over the 24 weeks, none of the diabetic animals developed anemia, as indicated by normal RBC, HGB and HCT values (Fig. 3D–F). Moreover, a significant increase in RBC (except for RBC in the 7-week-old females), HGB and HCT was observed in the 7- and 12-week-old diabetic animals, both in males and females compared to age-matched controls. These differences between healthy and diabetic individuals were particularly pronounced in 12-week-old females. The significant increase in RBC, HGB, and HCT values in 7- and 12-week-old *db/db* mice suggests a compensatory mechanism, possibly in response to hypoxia, or the influence of diabetes on erythrocyte turnover. The rise in RBCs level observed in hyperglycemia is commonly associated with insulin resistance, and it has been proven that insulin regulates erythropoiesis *in* vitro [52]. In contrast, we found no statistically significant differences (P > 0.05) between healthy and diabetic individuals in 24-week-old mice, suggesting that RBC, HGB and HCT in *db/db* mice do not differ from C57 controls of the same age despite progressive disease. It has also been postulated [52] that modulation of RBCs population dynamics may be a fundamental homeostatic response to various diseases. The results obtained in our study for the oldest animals may provide indirect evidence of this hemotopoietic homeostasis, which is primarily regulated by membrane-associated proteins [53]. Likewise, the elevated WBC levels observed in diabetic animals at 7 and 12 weeks of age suggest immune system activation due to disease progression. In contrast, the WBC levels at 24 weeks, which are comparable to those of healthy individuals, indicate the organism's efforts to achieve homeostasis (see Fig. 7 in Supplemental Materials).

### Analysis of changes in RBC surface topography in *db/db* mice using AFM

To analyze the surface and shape of erythrocytes, we employed the AFM technique. Fig. 4 illustrates the height-to-diameter ratio (N = 30, Fig. 4A), an example of a 3D topography image (Fig. 4B) and a sample height profile (Fig. 4C) for individual RBCs. All height and diameter measurements were performed on RBCs applied to the same surface, as determined by previous experiments [44,51]. The AFM data show that the majority of air-dried RBCs maintained their typical biconcave disc morphology, with diameters ranging from 4.0 to 4.7 µm. The height-to-diameter (h/d) ratio of fixed with 0.5 % glutaraldehyde RBCs, where height was measured at the peak of the profile (Fig. 4C), increased with the age of the mice in both C57 and *db/db* strains, with statistically significant differences observed in several cases. A difference in the *h/d* ratio (Fig. 4A') was observed with RBC aging for both diabetic animals as well as control, with slight changes of trends in 24 week-old mice. The RBC *h/d* ratio decreased in 24-week-old diabetic males compared to age-matched controls, while a slight, but not statistically significant (P *>* 0.05), increase was observed in 24-week-old diabetic females relative to their age-matched controls. Inversely proportional trends relative to the h/d ratio were observed in RBC membrane composition differences between 24-week-old males and females, particularly in the relative spectral contributions of protein secondary structures and aromatic side-chain–associated bands (Fig. 6C–E). The increase in the h/d ratio correlates with a decrease in the 1610 cm^−1^ aromatic band (phenyl-/aromatic side chains) and a reduction in the amide II/amide I ratio, indicating changes in protein secondary structure further analyzed in Section 3.4. This suggests that alterations in the h/d ratio are linked to protein degradation and to modifications in the local molecular environment of membrane proteins within the RBC membrane.

**Fig. 4:**
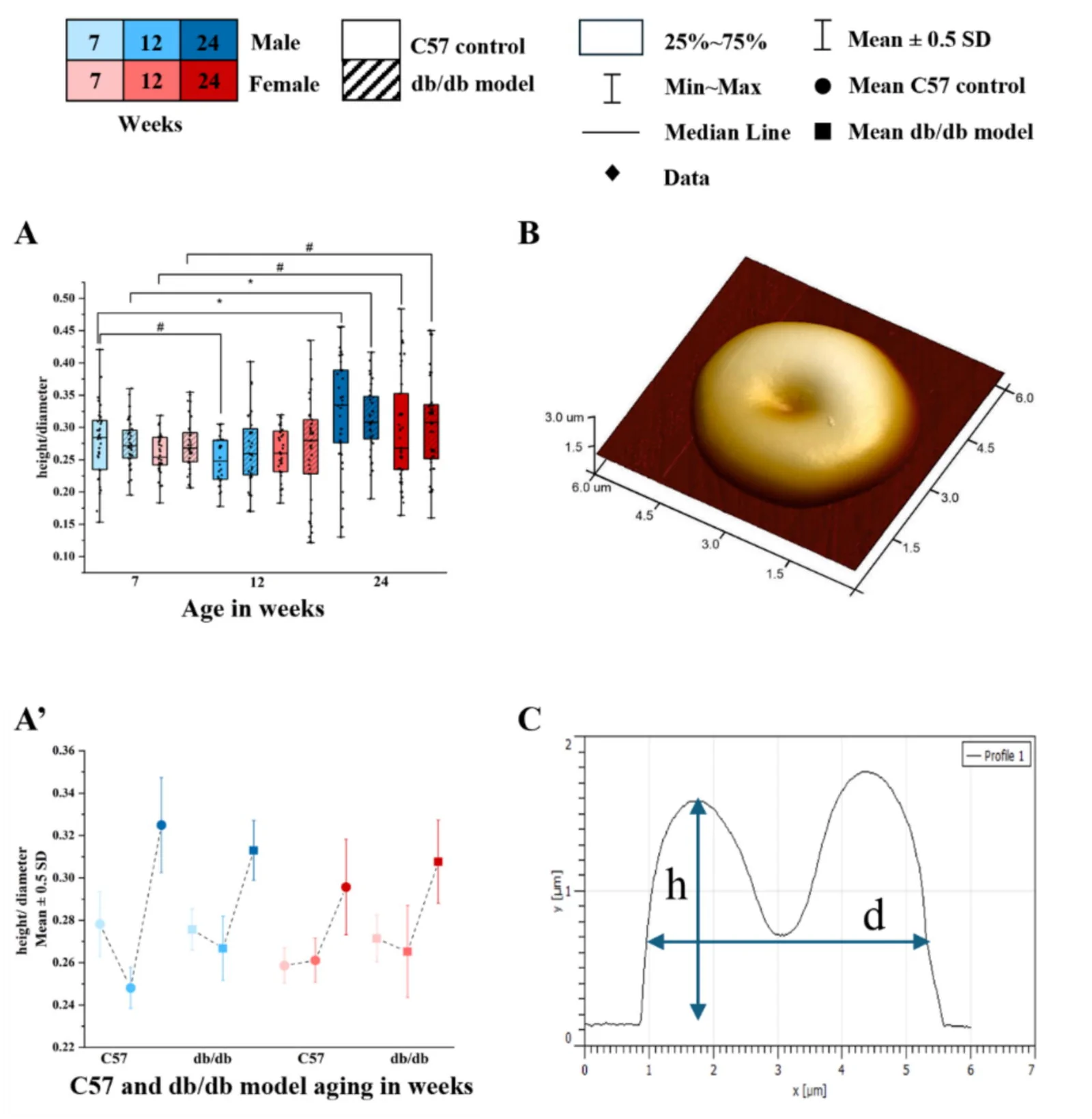
Changes in the height/diameter ratio (h/d) (A) during the progression of diabetes (at 7, 12, and 24 weeks of age) in male and female db/db mice, compared to C57 control mice, are illustrated. Data (A) is presented as box plots, showing the median, interquartile range, and min–max whiskers. Normality was assessed using the Shapiro-Wilk test, and statistical significance was determined by one-way ANOVA (#P < 0.05, *P < 0.01). Changes in the mean values of the height/diameter ratio (A’) over the course of diabetes progression (at 7, 12, and 24 weeks of age) in male and female db/db mice, compared to age-matched C57 controls, are also shown. The data distribution (A’) is presented as interval plots, with mean values and standard deviation whiskers. A dashed line connects the mean values to indicate the overall trend in the analyzed parameters from 7 to 24 weeks of age in both db/db and C57 control mice. Representative images of AFM topography in a 3D view (B) and topography cross-sections (C) of RBCs (h − height, d − diameter) are presented. The RBC samples were fixed with 0.5 % glutaraldehyde.

### Blood plasma from C57 and *db/db* mice

The impact of diabetes mellitus on dyslipidemia is illustrated in Fig. 5A-D. The diabetic animals exhibit notable lipoprotein abnormalities in their blood plasma, characterized by elevated total cholesterol (TC), elevated high-density lipoprotein (HDL) and elevated triglycerides (TG), particularly pronounced in 12-week-old mice. Additionally, both the 12- and 24-week-old diabetic mice show increased levels of low-density lipoprotein (LDL). Over the course of the study, TC and HDL levels were markedly elevated in *db/db* mice relative to control groups. The concentration changes for these parameters from 7 to 24 weeks were similar in both naturally aging and diabetic animals. The primary distinction between the diabetic and healthy individuals was a pronounced increase in these parameters observed in *db/db* mice from 12 to 24 weeks. Consequently, significant differences in TC and HDL levels were noted in the oldest diabetic mice compared to healthy control animals.

**Fig. 5:**
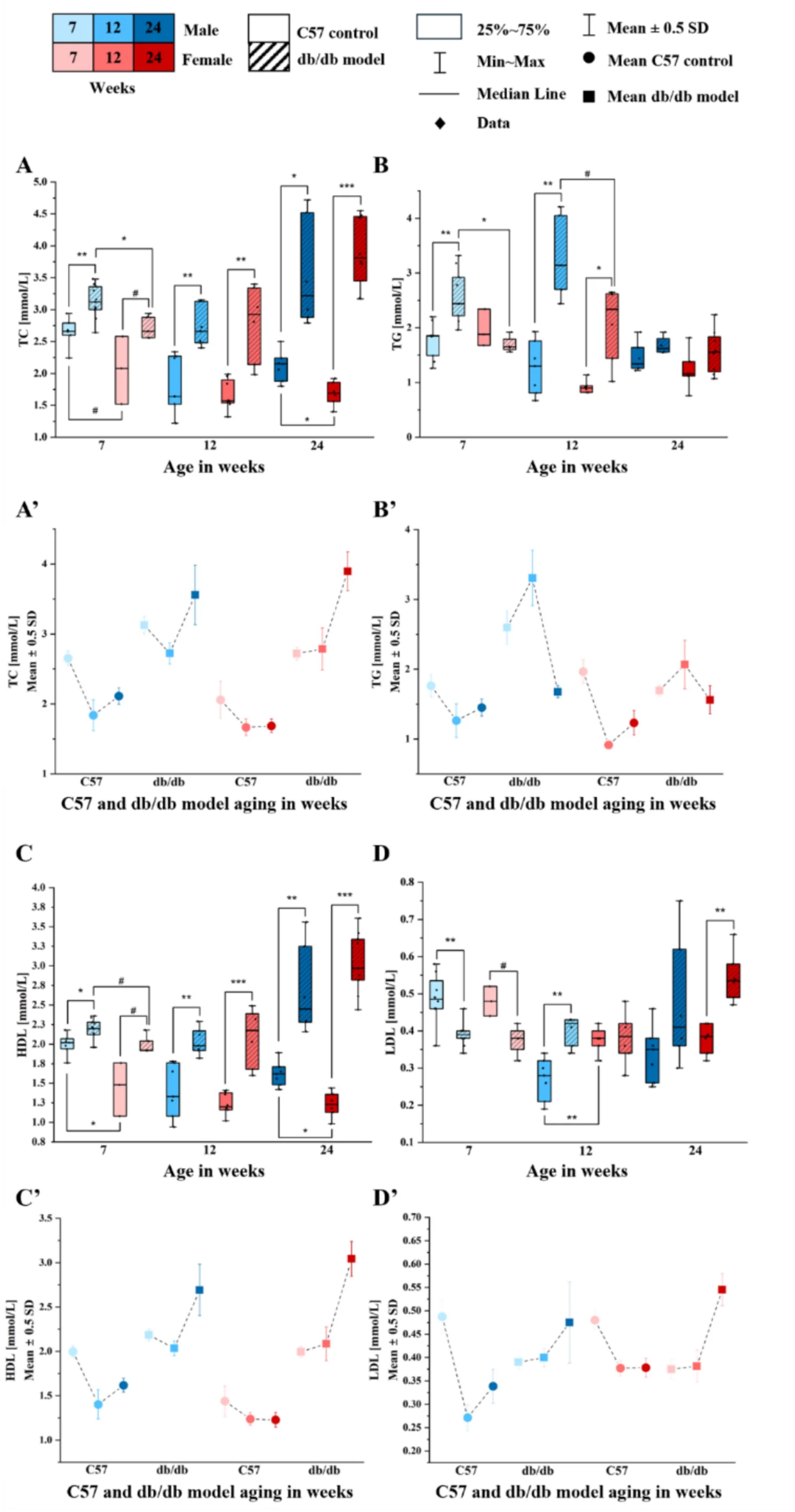
Changes in cholesterol, TC (A), triglycerides, TG (B), LDL (C), and HDL (D) levels in blood plasma during the progression of diabetes (at 7, 12, and 24 weeks of age) in male and female db/db mice compared to C57 control mice are illustrated. The data (A-C) are presented as box plots displaying the median**,** interquartile range, and min–max whiskers. The Shapiro-Wilk test was used to assess normality. Statistical significance was determined using the Mann-Whitney test (#P < 0.05, *P < 0.01, **P < 0.001, ***P < 0.0001). Changes in in cholesterol, TC (A’), triglycerides, TG (B’), LDL (C’), and HDL (D’) levels in blood plasma over the course of diabetes progression (at 7, 12, and 24 weeks of age) in male and female db/db mice, compared to age-matched C57 controls, are also depicted. The data distribution (A’-D’) is presented as interval plots, where the mean values are shown with standard deviation whiskers. A dashed line connects the mean values to indicate the general trend in the analyzed parameters from 7 to 24 weeks of age in both db/db and control C57 mice. Abbreviations: TC – cholesterol, TG – triglycerides, LDL – Low-Density Lipoprotein, HDL – High-Density Lipoprotein.

Throughout the study, statistically significant differences in TG levels were documented in 7-week-old male and 12-week-old male and female diabetic mice when contrasted with age-matched control. The remaining *db/db* mice did not demonstrate significant differences from healthy controls. Moreover, the mean TG concentrations during the study period were distinctly different between healthy and diabetic animals (Fig. 5). When TG levels decreased in healthy individuals, they simultaneously increased in diabetic mice, and vice versa. This suggests that regulation of lipid metabolism is controlled differently in control and diabetic animals as they age. In 24-week-old *db/db* animals, despite a slightly, but not significantly (P *>* 0.05), elevated TG level, the concentration does not indicate any pathological conditions.

Phosphorus (P), creatinine (CR) and lactate dehydrogenase (LDH) levels were also quantified to evaluate potential metabolic and renal complications, as well as tissue damage commonly associated with diabetes (Supplemental Fig. S7B–D) [54,55]. Elevated phosphorus and creatinine levels were observed in all *db/db* mice, indicating potential renal impairment, as both are key markers of renal function. In contrast, LDH levels did not show any statistically significant increase (P *>* 0.05), suggesting the absence of substantial cellular damage in the diabetic mice.

### Vibrational spectroscopy in analysis of diabetic RBC alterations

To explore the biochemical composition and structural alterations in RBCs and RBC membranes from C57 and *db/db* mice, both RS and IR spectroscopies were employed (see Fig. 1). These techniques provided detailed information on lipid and protein dynamics, highlighting significant changes related to diabetes, age and gender.

#### General RBC membrane alteration – FT-IR and RS

The analysis of FT-IR spectra (Fig. 6A–C) shows mainly changes in lipid unsaturation (3012/2925), cholesterol ester content (1161/2925) and secondary protein structure alterations (1544/1651) in the RBC membranes. The 3012 cm^−1^ band corresponds to C–H stretching in unsaturated lipids [56,57], while the 2925 cm^−1^ band represents the C–H stretching in saturated lipids [58,59]. The 3012/2925 ratio provides a measure of lipid unsaturation relative to total lipids. Interestingly, a negative correlation between blood plasma glucose and this lipid unsaturation ratio was found (R^2^ = 0.73, P < 0.05), suggesting that lipid-related alterations in RBC membranes reflect the direct metabolic consequences of hyperglycemia (Supplemental Fig. S8). The 1161 cm^−1^ band reflects cholesterol ester content [60,61], while the 2925 cm^−1^ band indicates CH_2_ stretching, making this ratio useful for assessing the balance between cholesterol esters and overall lipids content. The 1544 cm^−1^ band corresponds to amide II vibrations [62,63], while the 1651 cm^−1^ band reflects amide I content [19,64]. This ratio highlights alterations in protein secondary structure.

Raman spectroscopy was applied using a 532 nm laser line excitation (488 nm excitation presented in Supplementary Materials), with specific focus on bands that reflect triglycerides (1264/2853), spectral contributions from aromatic amino-acid side chains (1610/1447) and lipid esterification (1743/2853) (Fig. 6D–F). The 1610 cm^−1^ band represents C=C stretching in aromatic rings, typically dominated by phenylalanine, tyrosine or tryptophan residues in proteins [65,66] and is therefore interpreted here as an aromatic side-chain–associated spectral marker rather than a direct measure of absolute amino-acid content. The 1447 cm^−1^ band, linked to C–H bending, is common to both lipids and proteins, aiding in the analysis of protein–lipid interactions [44]. TG are not intrinsic constituents of the erythrocyte membrane, yet a small exchangeable pool of neutral lipids including trace amounts of TGs can transiently associate with the cell surface through continuous lipid exchange with plasma lipoproteins [67,68]. The TG-related Raman bands detected in the membrane fraction therefore most likely originate from this plasma-derived, membrane-associated pool rather than from membrane-embedded triglycerides. As all samples were processed under identical conditions, the attenuated TG signal in RBCs from db/db mice reflects a genuine perturbation in lipid exchange or handling, consistent with the alterations observed in plasma.

The combined RS and IR data revealed substantial lipid alterations as well as alterations in proteins in the RBC membranes of *db/db* mice as opposed to controls, with variations influenced by age and gender. TG levels in RBC membranes, increased with age in both healthy and diabetic mice, with significantly higher values in females across all groups. However, *db/db* mice, particularly males, showed a marked reduction in TG levels compared to age-matched controls. This observation aligns with the results of TG levels in blood plasma, which fluctuated between diabetic and healthy animals. *Db/db* mice exhibited elevated TG concentrations in blood plasma, while simultaneously showing reduced TG levels in RBC membranes. This suggests a distinct regulation of lipid metabolism in *db/db* mice versus control animals. The higher TG concentration in blood plasma may indicate impaired lipid clearance or increased lipolysis, leading to an accumulation of TGs in circulation. Meanwhile, the lower TG levels in RBC membranes suggest that TGs may not be efficiently incorporated into or utilized by RBCs, reflecting an imbalance in lipid transport and distribution.

Lipid unsaturation showed a consistent decrease as individuals grow older in both *db/db* and control mice. This decrease in unsaturation was more pronounced in diabetic animals, suggesting increase in saturated lipids, which can contribute to decreased membrane fluidity. Additionally, diabetic mice exhibited lower levels of unsaturation compared to age-matched controls, with the disparity becoming more pronounced in older animals. Lipid esterification, including cholesterol esters, remained relatively stable over time, with the highest increase in 12- and 24-week diabetic males and 24-week healthy and diabetic females (Fig. 6F).

**Fig. 6:**
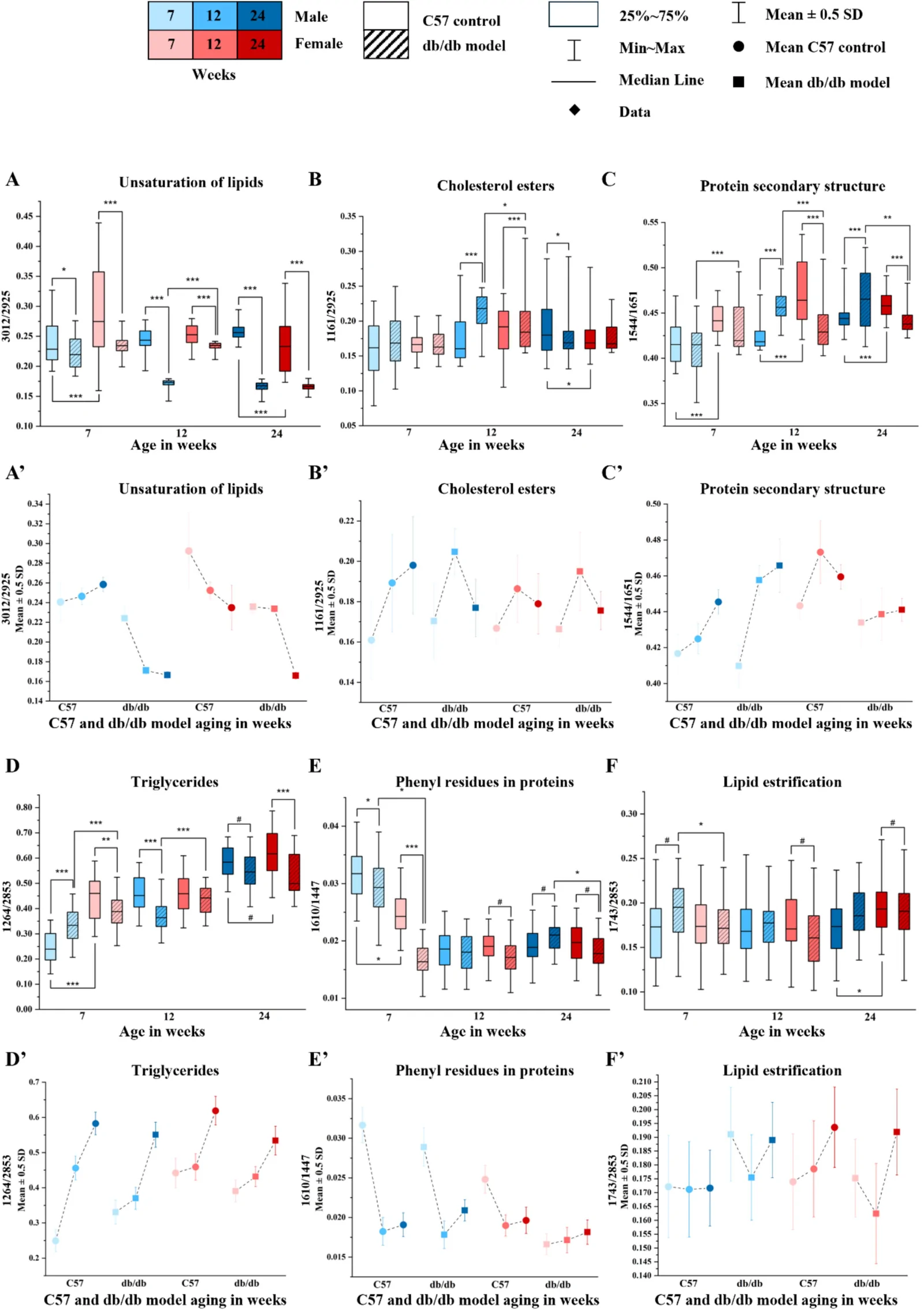
The ratios of the integral intensities of the selected (A-C) IR bands and (D-F) Raman bands (532 nm laser excitation) obtained from RBC membranes of 7, 12, and 24-week-old C57BL/6J and db/db mice. The integration regions for the FT-IR transmission bands include: C–O–C stretch at 1161 cm^−1^ (1191–1144 cm^−1^), amide II at 1544 cm^−1^ (1560–1502 cm^−1^), amide I at 1651 cm^−1^ (1687–1605 cm^−1^), CH_2_ asymmetric stretch at 2925 cm^−1^ (2942–2918 cm^−1^), and =C–H stretch at 3012 cm^−1^ (3006–3022 cm^−1^); The integration regions for the Raman bands include: scissoring and bending vibrations of CH_2_ and CH_3_ at 1743 cm^−1^ (1709–1760 cm^−1^), symmetric CH_2_ stretch at 2853 cm^−1^ (2836–2862 cm^−1^), C–H bending at 1447 cm^−1^ (1419–1479 cm^−1^), =C–H bending mode at 1264 cm^−1^ (1220–1280 cm^−1^), and C=C stretch in aromatic rings at 1610 cm^−1^ (1592–1618 cm^−1^). Normality was assessed using the Shapiro-Wilk test. Data are presented as box plots (median, min–max whiskers, and interquartile range), and significance was calculated using one-way ANOVA (#P < 0.05, *P < 0.01, **P < 0.001, ***P < 0.0001). Changes in mean FT-IR transmission ratios (A'-C') and Raman scattering (RS) ratios (D’-F’) were calculated with the progression of diabetes (7, 12, and 24 weeks) in male and female db/db mice compared to age-matched C57 controls. The same ratios for 488 nm excitation are presented in Supplemental Fig. S9.

The lipid membrane analysis together with blood plasma and mice weight analysis suggests gender-specific differences in lipid processing. Gender-specific differences in lipid metabolism were observed, with female diabetic mice consistently showing higher lipid levels compared to males. This suggests that lipid processing may be regulated differently between sexes, especially in older females, who exhibited higher weight gain and greater lipid accumulation.

Taken together, these findings indicate that although db/db mice develop a severe diabetic and dyslipidemic phenotype, their plasma lipid profile does not fully reflect the pattern usually observed in human T2DM, where elevated TG, reduced HDL and an increased atherogenic LDL burden are common [69]. In our cohort, db/db mice displayed persistently high total and HDL cholesterol, only moderate or transient increases in TG and relatively low LDL levels, indicating a model-specific pattern of lipid metabolism and cardiovascular risk that warrants caution when extrapolating lipid-associated RBC alterations to human patients. Although the present study focuses exclusively on the db/db mouse model of T2DM, this choice reflects a deliberate strategy to enable deep, internally consistent phenotyping across sex and age within a single, well-controlled cohort, integrating haematological, biochemical, AFM, FT-IR and Raman datasets. The db/db model, characterised by a stable and well-defined diabetic metabolic milieu, provides a powerful initial platform for delineating spectrochemical signatures associated with chronic hyperglycaemia, dyslipidaemia, oxidative stress and protein lipid remodelling of the erythrocyte membrane processes that are broadly shared across many forms of T2DM. Consequently, the core vibrational markers identified here are expected to extend beyond this specific model. Building on this foundation, the next essential step will be to validate these candidate markers in human diabetic cohorts and to further elucidate their mechanistic underpinnings in additional, biologically distinct T2DM models (e.g., ob/ob mice). Such sequential validation will be critical for establishing the generalisability and translational relevance of the spectrochemical framework introduced in this study.

#### Detailed analysis of protein content alterations − protein misfolding and aggregation

Multivariate analysis was performed to enhance the visualization of diabetes induced structural alterations at 24 weeks. The oPLS-DA model for FT-IR RBCs membranes was able to discriminate *db/db* from C57 membranes with a LOAO CV classification error of 14 % and 5 % for females and males, respectively. Fig. 7A-B show that *db/db* mice samples were almost perfectly classified accordingly, while a higher false positive ratio in females explains its slightly, but not significantly (P > 0.05), higher classification error compared with males. This can also be visualized in the AUROC curve from Fig. 7C, with values of 0.938 and 0.986 for females and males, respectively. Still, statistical significance (P < 0.05) of models from both genders was confirmed via permutation testing as displayed in Fig. 7D.

**Fig. 7:**
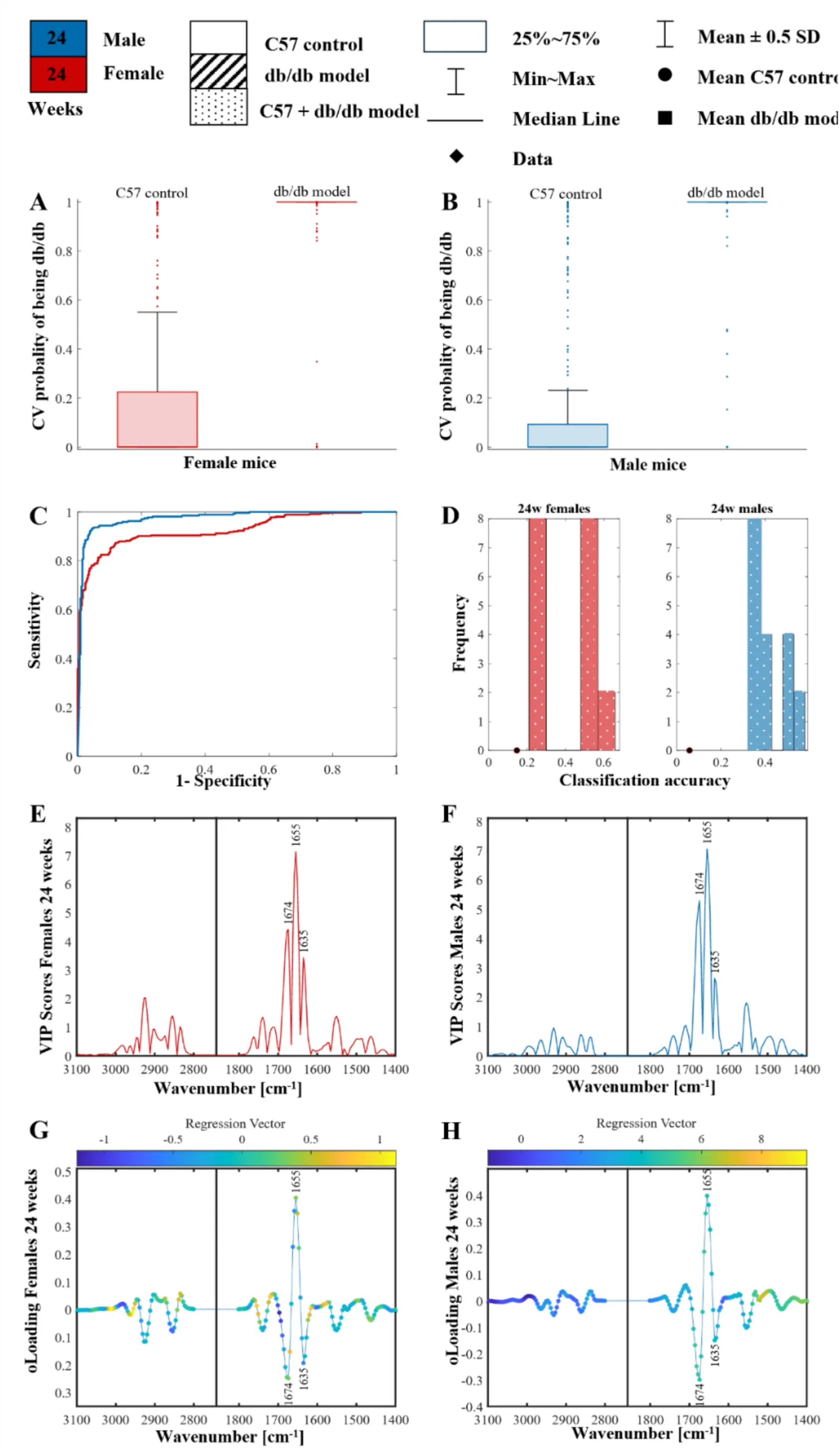
Boxplot of the CV probability of being classified as db/db of the oPLS-DA for female (A) and male (B) according to FT-IR RBCs membranes. AUROC curve of the diabetic classification for females (red) and males (blue) (C). Histogram of CV Errors of the classification of C57 control vs. db/db obtained from permutation testing of the oPLS-DA model (D) for females (red) and males (blue). The black dot indicates the error of the actual, unpermuted data, which falls outside the main distribution of permuted values, highlighting the statistical significance P < 0.05) of the oPLS-DA model compared to random chance. VIP Scores of the oPLS-DA model for females (E) and males (F). Orthogonalized loading coloured according to the regression vector for females (G) and males (H). (For interpretation of the references to colour in this figure legend, the reader is referred to the web version of this article.)

The first orthogonalized loading (Fig. 7G-H), which captures the main covariance, can be explained by protein secondary structures alterations. Second derivative was included in the preprocessing, so local maxima are actually local minima and vice versa. Therefore, *db/db* mice presents mainly a higher β-turn (1674 cm^−1^) content. In addition, band at 1635 cm^−1^ also suggests an increase in antiparallel β-sheet (1631 cm^−1^) and H-bonded β-turn (1639 cm^−1^) content. At the same time, α-helix (1655 cm^−1^) was negatively associated with *db/db* mice. These finding are reinforced when analyzing their corresponding VIP scores (Fig. 7E–F), as the same variables associated with the same secondary protein structures were found to be key in the classification model. Thus, a tendency towards reduced α-helix content and increased β structures was observed in the diabetic model for both female and male. Overall, this multivariate analysis emphasizes the long-term effects on RBCs membranes of the exposure to the diabetic disease.

A corresponding oPLS-DA model was developed for 488 nm Raman spectroscopy, achieving a similar discriminatory power with a LOAO CV classification error of 9 % and 13 % and a AUROC of 0.967 and 0.931 for females and males, respectively. Statistically significant separation (P < 0.05, see Fig. 8D) between diabetic and control groups for RBCs membranes was observed primarily in the C–H stretching region, with limited contribution from the protein-related spectral domain as depicted in the VIP scores and loadings (Fig. 8E–H). Main discriminatory bands arise from CH_2_ (2853 cm^−1^) and CH_3_ (2936 cm^−1^) stretching of membrane lipids. This highlights that FT-IR and Raman are complementary techniques, both useful for the molecular staging of diabetes but focusing on secondary protein structures and lipid content, respectively.

**Fig. 8:**
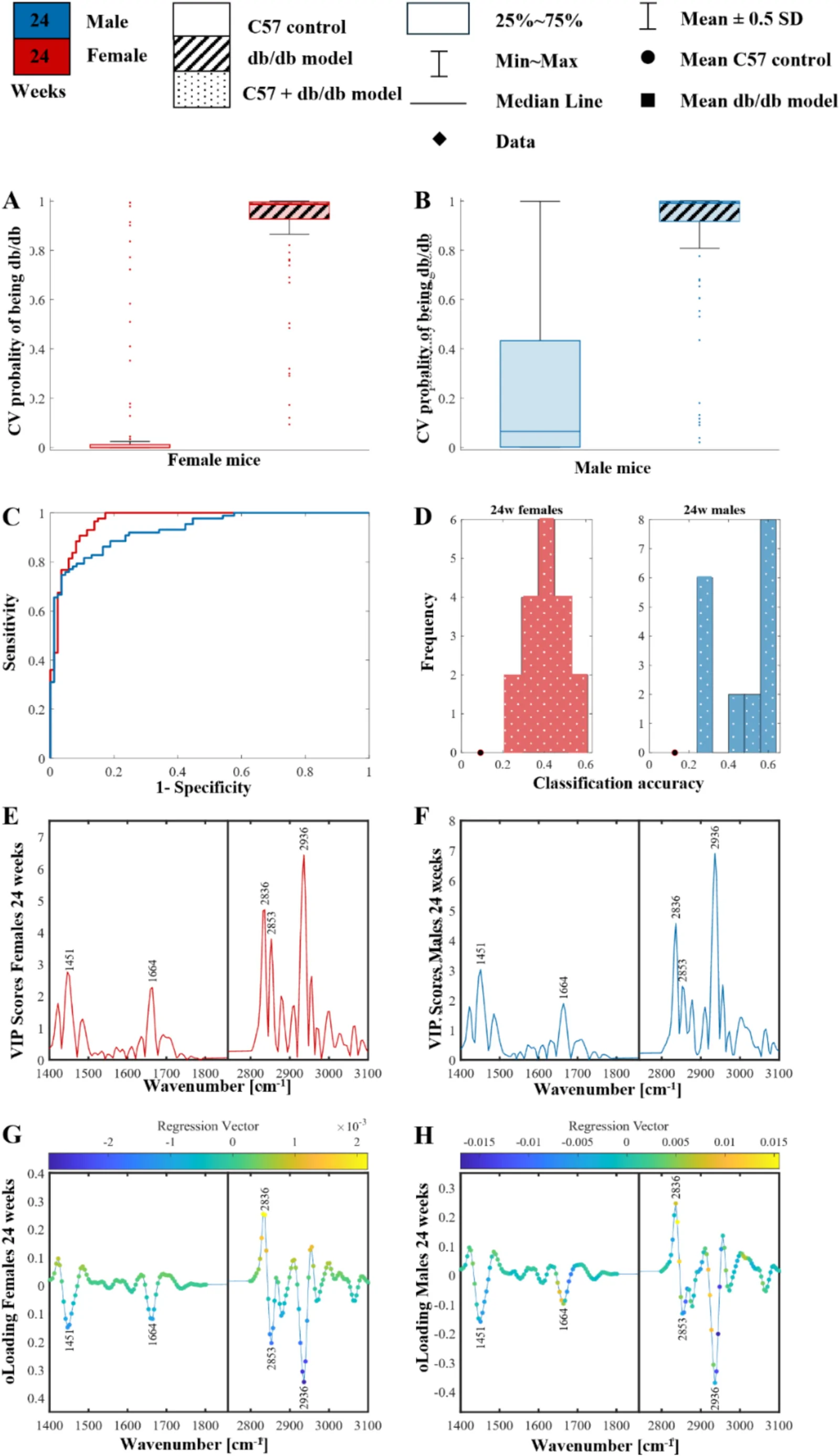
Boxplot of the CV probability of being classified as db/db of the oPLS-DA for female (A) and male (B) according to Raman RBCs membranes. AUROC curve of the diabetic classification for females (red) and males (blue) (C). Histogram of CV Errors of the classification of C57 control vs. db/db obtained from permutation testing of the oPLS-DA model (D) for females (red) and males (blue). The black dot indicates the error of the actual, unpermuted data, which falls outside the main distribution of permuted values, highlighting the statistical significance P < 0.05) of the oPLS-DA model compared to random chance. VIP Scores of the oPLS-DA model for females (E) and males (F). Orthogonalized loading coloured according to the regression vector for females (G) and males (H). (For interpretation of the references to colour in this figure legend, the reader is referred to the web version of this article.)

Main effects of diabetes on RBCs membranes appear to be common for both females and males, with similar changes on protein secondary structure and lipid content. Loadings and VIP scores showed gender-independent profiles for both FT-IR and Raman scattering. However next we performed two additional oPLS-DA to try to enhance visualization of gender differences. Structural discrepancies between the RBCs membranes of males and females at 24 weeks were observed using FT-IR. Notably, although C57 mice could be classified according to gender using oPLS-DA (Supplemental Fig. S11A, C) with an AUROC of 0.876, the separation between these two groups was superior in *db/db* mice (Supplemental Fig. S11B, C) with an AUROC of 0.964, suggesting that diabetes effects are, at least in part, gender-specific. Main assigned differences in protein secondary structure between females and males were assigned to aggregative structures such as hydrated β-sheet (1612 cm^−1^) and H-bonded antiparallel β-sheets (1700 cm^−1^) as visualized in the VIP scores and loading in Supplemental Fig. S11F, H. These features were positively correlated with males. Thus, although protein structural alterations are common for both males and females, mainly arising from non-aggregative secondary structures, the severity of the diabetes effect on aggregative structures may be different depending on the gender. No statistically significant differences (P > 0.05) between males and females’ RBCs membranes at 24 weeks were found using Raman.

The further detailed evaluation of secondary protein structure alterations in RBC membranes was conducted by comparing relative absorption levels in spectral bands corresponding to selected secondary structures between healthy and *db/db* mice. The examined bands were categorized into two groups: non-aggregative and aggregative structures. Supplemental Figs. S2, S5 and S6 present the most important regions taken into analysis, for RS with 532 nm and 488 nm as well as FT-IR, respectively.

##### Changes in secondary structures of non-aggregative RBC membrane proteins

Non-aggregative structures included α-helix (FT-IR: 1658 cm^−1^, RS: 1656 cm^−1^), antiparallel β-sheet (FT-IR: 1631 cm^−1^, RS: 1690 cm^−1^), β-turn (FT-IR: 1674 cm^−1^, RS: 1670 cm^−1^) and β-sheet (FT-IR: 1689 cm^−1^). Given the critical role of sample thickness in FT-IR measurements, normalization was performed using the most prominent band, α-helix, as the reference.

For FT-IR analysis, three band ratios were assessed to determine the relative content of non-aggregative secondary protein structures and their variations were presented in Fig. 9A, C. The first ratio, 1631/1658, represented the relative content of antiparallel β-sheet to α-helix. As shown in Fig. 9A-D, a significant increase in antiparallel β-sheet content was observed in the oldest *db/db* mice compared to controls, whereas differences were less pronounced in younger groups. Additionally, at 7 weeks of age, males exhibited a higher relative content of antiparallel β-sheet, a difference that diminished with age. RS results presented in Fig. 9B confirmed this trend, as evidenced by an increase in the 1690/1656 ratio in the *db/db* group across all age groups, rather than being restricted to older animals.

**Fig. 9:**
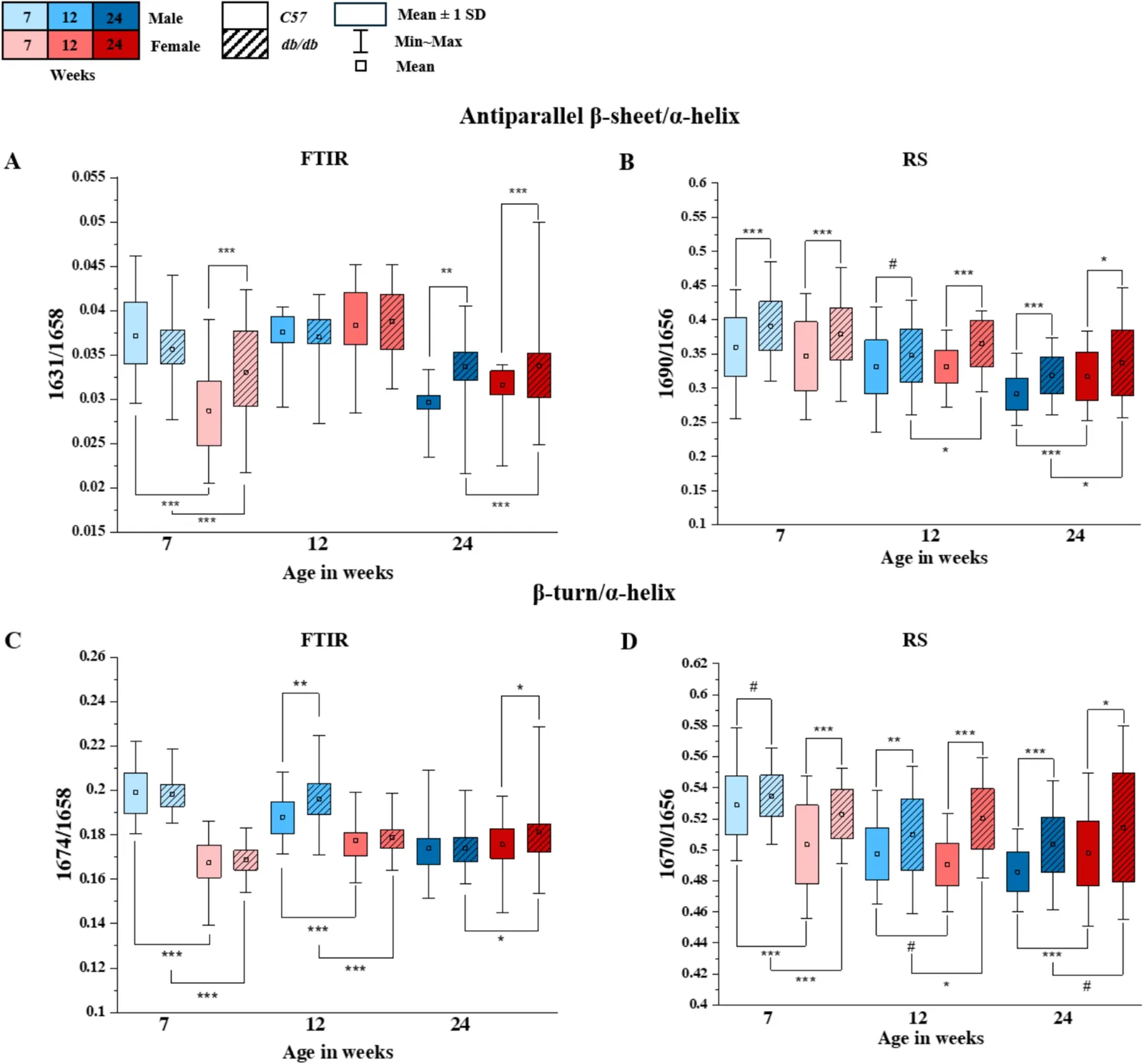
The ratios of integral intensities for selected FT-IR (A, C) and RS (B, D) bands (532 nm laser excitation) were analyzed to assess alterations in the secondary structures of RBC membrane proteins for C57BL/6J and db/db Mice of Both Sexes (Male/M: Blue, Female/F: Red) at 7, 12, and 24 Weeks of Age. FT-IR bands included 1631/1658 cm^−1^ (A) and 1674/1658 cm^−1^ (C), reflecting changes from antiparallel β-sheets to α-helices and from β-turns to α-helices, respectively. Similarly, RS bands included 1690/1656 cm^−1^ (B) and 1670/1656 cm^−1^ (D), corresponding to analogous structural transitions. Normality was assessed using the Shapiro-Wilk test. Data are presented as box plots (mean, min–max whiskers, and standard deviation), and significance was calculated using one-way ANOVA (#P < 0.05, *P < 0.01, **P < 0.001, ***P < 0.0001). (For interpretation of the references to colour in this figure legend, the reader is referred to the web version of this article.)

The second ratio, 1674/1658, corresponded to the relative content of β-turn to α-helix. The results demonstrated a higher β-turn content in *db/db* mice across all tested groups. Furthermore, at 7 weeks of age, males exhibited higher β-turn levels than females, a difference that disappeared with age. RS analysis corroborated this trend, showing an increased 1670/1656 ratio in *db/db* mice relative to controls across all age groups. Similar to FT-IR results, younger males had higher β-turn content than females, but this trend reversed in older animals.

The 1689/1658 ratio describes the relative content of β-sheet to α-helix. Fig. 10A illustrates that β-sheet content was elevated in *db/db* mice across nearly all tested groups, with differences becoming more pronounced with age. Additionally, at 7 weeks of age, males exhibited higher β-sheet content, but this difference diminished over time. These findings highlight significant structural modifications in RBC membrane proteins associated with diabetes, emphasizing the potential impact of disease progression, age, and gender even on protein conformation of non-aggregative protein structures.

**Fig. 10:**
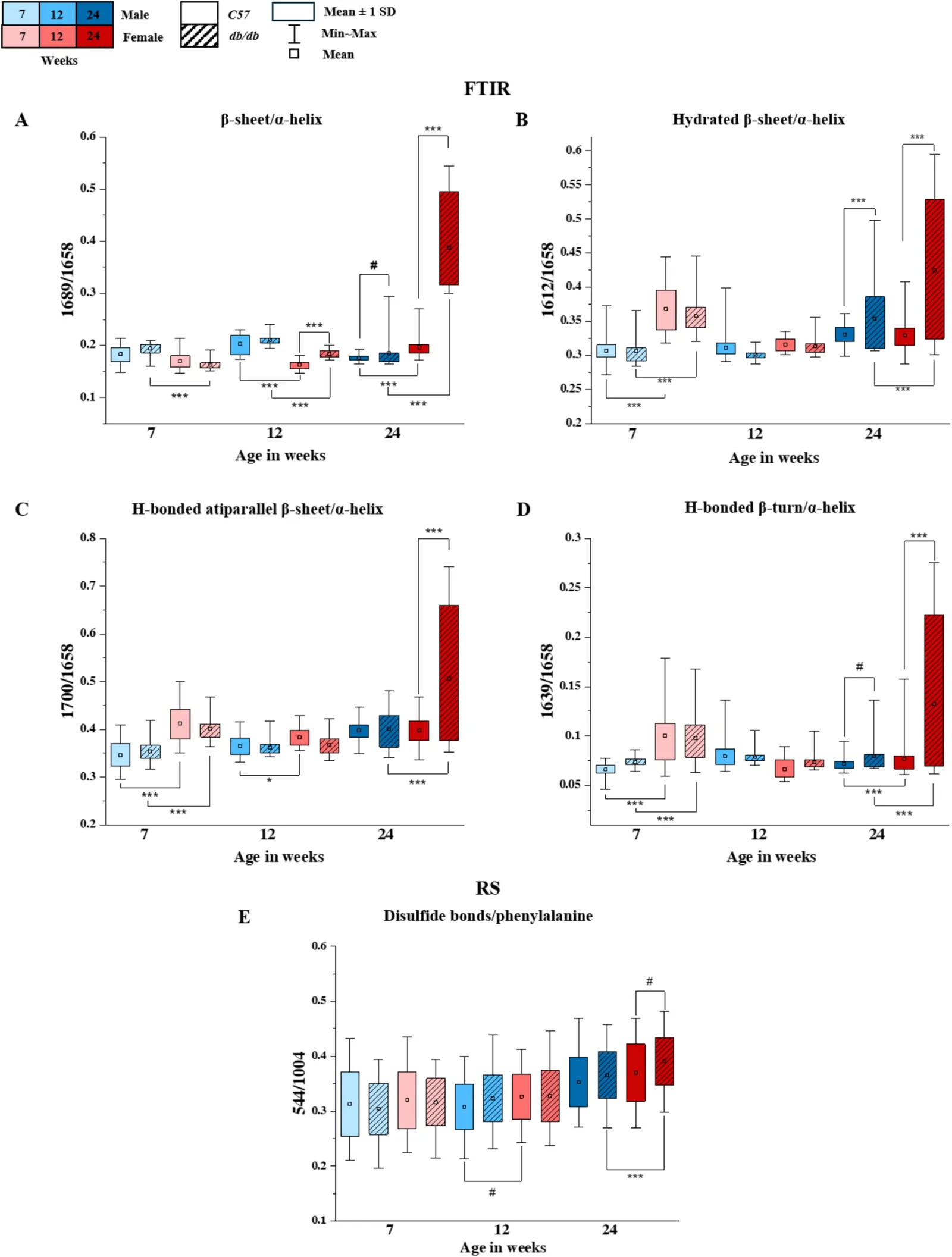
The ratios of integral intensities for selected FT-IR (A-D) and RS (E) bands (532 nm laser excitation) were analyzed to assess alterations in the secondary structures of RBC membrane proteins for C57BL/6J and db/db Mice of Both Sexes (Male/M: Blue, Female/F: Red) at 7, 12, and 24 Weeks of Age. The ratio of the integral intensities for the FT-IR transmission bands include: 1689/1658 cm^−1^ (A), 1612/1658 cm^−1^ (B), 1700/1658 cm^−1^ (C) and 1639/1658 cm^−1^ (D) showing alterations in secondary structures of RBC proteins, i.e. β-sheet to α–helices, hydrated β-sheet to α–helices, H-bonded antiparallel β-sheet to α–helices and H-bonded β-turns to α–helices, respectively. The ratio of the integral intensities for the RS bands include: 544/1004 cm^−1^ (E) showing alterations in tertiary structures of RBC proteins and corresponding to changes in disulfide bonds to phenylalanine. Normality was assessed using the Shapiro-Wilk test. Data are presented as box plots (mean, min–max whiskers, and standard deviation), and significance was calculated using one-way ANOVA (#P < 0.05, *P < 0.01, **P < 0.001, ***P < 0.0001). The ratios for RS 488 nm excitation are presented in Supplemental Fig. S10. (For interpretation of the references to colour in this figure legend, the reader is referred to the web version of this article.)

##### Changes in secondary structures of aggregative RBC membrane proteins

Aggregative structures arise from stress-induced modifications of non-aggregative ones, and their presence in the cell membrane may indicate improper protein folding or oxidative stress. The following bands were classified as aggregative: hydrated β-sheet (FT-IR: 1612 cm^−1^), H-bonded β-turn (FT-IR: 1639 cm^−1^) and H-bonded antiparallel β-sheets (FT-IR: 1700 cm^−1^). Data normalization to α-helix was also applied.

The first analyzed band ratio, 1612/1658, represented the relative content of hydrated β-sheet to α-helix. As shown in Fig. 10B, the most pronounced differences were observed in the 24-week group, where *db/db* mice exhibited higher hydrated β-sheet content than controls. In terms of sex differences, all female groups showed slightly, but not significantly (P > 0.05), higher values compared to their male counterparts.

The 1639/1658 ratio, which corresponds to the relative content of H-bonded β-turn to α-helix (Fig. 10D), remained relatively stable across all groups except at 24 weeks, where a substantial increase was observed in *db/db* females. A significant rise was also noted in 24-week *db/db* males. The 1700/1658 ratio, which indicated the relative content of H-bonded antiparallel β-sheets to α-helix (Fig. 10C), remained consistent across all groups, with a slight, but not statistically significant (P > 0.05), age-related increase and marginally higher values in females. However, at 24 weeks, *db/db* females exhibited a pronounced increase, with a significant rise also observed in *db/db* males at the same age. These findings suggest that diabetes and aging contribute to stress-induced structural modifications in RBC membrane proteins, with notable sex-dependent variations.

##### Changes in tertiary structures of proteins

Tertiary protein structure alterations in RBC membranes were assessed by analyzing the 544/1004 Raman intensity ratio across age, sex and diabetic status. The 544 cm^−1^ band (disulfide bonds) reflects protein folding integrity, while 1004 cm^−1^ (phenylalanine) served as an internal standard. Given the critical role of disulfide bonds in preserving protein conformation, this ratio provides valuable insights into structural disruptions linked to pathological conditions. No statistically significant differences (P > 0.05) were observed between healthy and diabetic groups, except for elevated ratios in 24-week-old diabetic females (Fig. 10E). An age-dependent increase in this ratio was noted in both sexes, with females consistently showing higher values, suggesting greater structural stability or differential regulation. Although aromatic side-chain–associated signals (Fig. 6E) do not directly indicate tertiary structure changes or alterations in amino-acid composition, their variation may reflect conformational shifts and/or changes in the local molecular environment of aromatic residues (e.g. packing, hydration and lipid–protein interactions). The relative intensity of this aromatic band declined with age in males, particularly from 7 to 12 weeks, and was generally higher in males than in females. Notably, diabetic females showed a marked reduction compared to controls, consistent with diabetes-related alterations in the abundance and/or microenvironment of aromatic-amino-acid–containing proteins and with RBC membrane remodelling.

#### Multivariate analysis of RBC – Fast diagnostics on intact RBC

Considering the entire composition of RBCs, analyzes of the relative content of specific secondary protein structures were conducted in parallel to those performed for RBC membranes (Fig. 11). However, these measurements were performed exclusively using FT-IR spectroscopy, as Raman spectroscopy would have resulted in resonance from hemoglobin, preventing a comprehensive analysis of all cytosolic proteins.

**Fig. 11:**
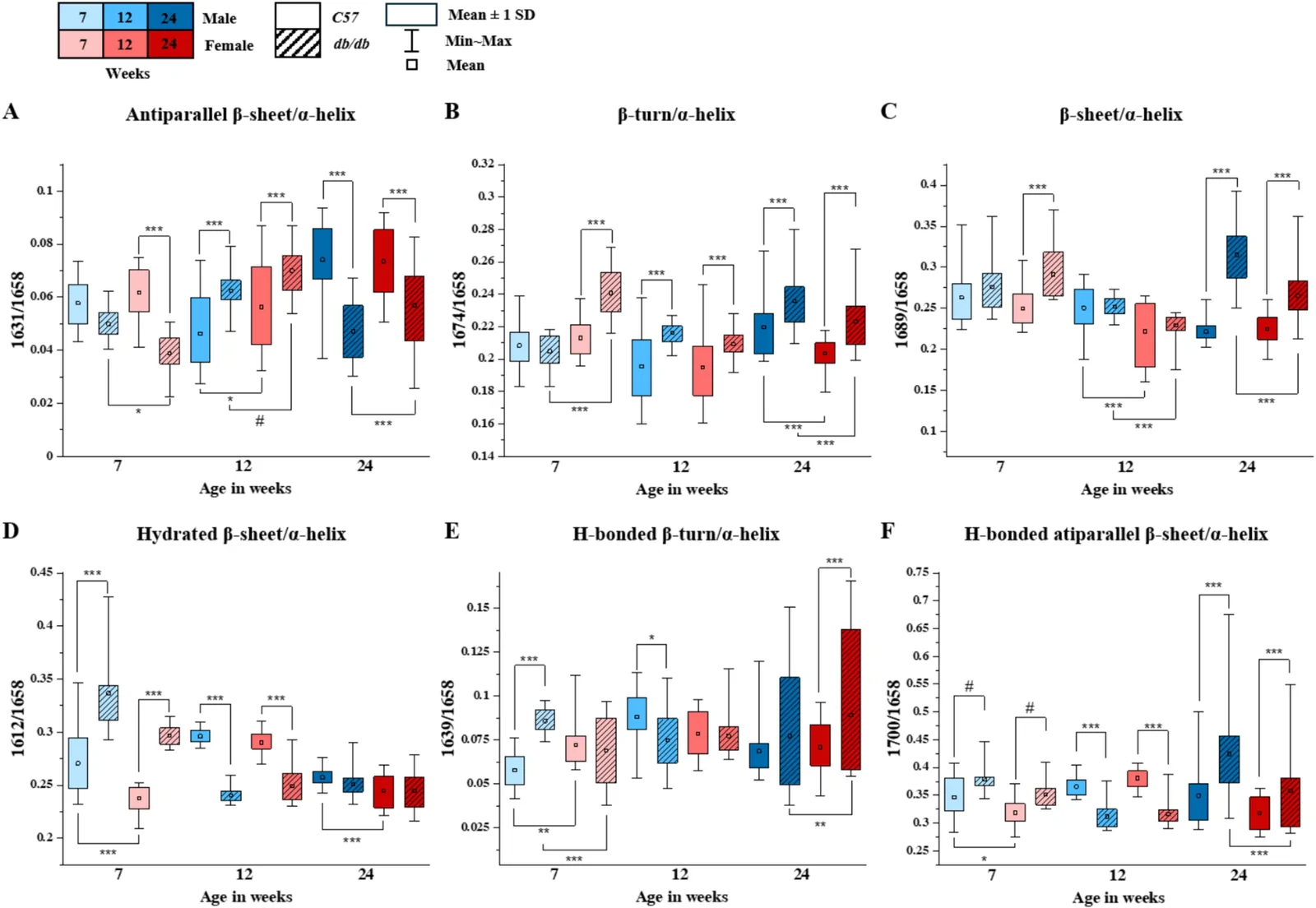
The ratios of integral intensities for selected FT-IR (A-F) were analyzed to assess alterations in the secondary structures of whole RBC proteins for C57BL/6J and db/db mice of both sexes (Male/M: Blue, Female/F: Red) at 7, 12, and 24 weeks of age. The top row included band ratios of 1631/1658 cm^−1^ (A), 1674/1658 cm^−1^ (B), and 1689/1658 cm^−1^, reflecting changes from antiparallel β-sheets to α-helices and from β-turns to α-helices and β-sheet to α–helices, respectively. The bottom row included band ratios of 1612/1658 cm^−1^ (D), 1639/1658 cm^−1^ (E) and 1700/1658 cm^−1^ (F), showing relative alterations in secondary structures of RBC proteins in hydrated β-sheet to α–helices, H-bonded β-turns to α–helices and H-bonded antiparallel β-sheet to α–helices, respectively. Normality was assessed using the Shapiro-Wilk test. Data are presented as box plots (mean, min–max whiskers, and standard deviation), and significance was calculated using one-way ANOVA (#P < 0.05, *P < 0.01, **P < 0.001, ***P < 0.0001). (For interpretation of the references to colour in this figure legend, the reader is referred to the web version of this article.)

First, PLS regression was employed to explore possible links between RBCs FTIR spectra and typical blood biochemical parameters, finding a significant correlation (R^2^ = 0.60, P < 0.05) between plasma blood glucose and RBCs spectral signature (Supplemental Fig. S12). Next, this time SVM-DA was used to discriminate *db/db* from C57 whole RBCs at 24 weeks, obtaining a LOAO CV classification error of 25 % and 21 % for females and males, respectively (Supplemental Table S2). Therefore, model performance was reduced compared to that obtained using RBC membrane spectra. This hints that, in whole RBCs, although a clear classification trend is observed, inter-animal variability masks diabetes-related spectral changes. However, when venetian blinds was used instead for CV, errors for both genders decreased to below 5 %. This suggests that increasing the number of mice included in the study would accomplish to model this inter-animal variability and significantly improve the performance of the discrimination models.

Since SVM-DA is a black-box model, a more detailed assessment of diabetes-induced alterations in protein secondary structure within whole RBCs was performed by directly comparing the relative absorbance of spectral bands associated with specific secondary structures between healthy and *db/db* mice.

The first analyzed ratio, 1631/1658, revealed that the relative content of antiparallel β-sheets increased with age in both males and females, with lower values observed in *db/db* mice in the youngest and oldest groups. A slight trend, with statistical significance at the level above 0.05, suggested a higher content in females compared to males. The 1674/1658 ratio, showed that the relative β-turn content remained generally stable across all groups, with a slight, but not statistically significant (P > 0.05), increase in 24-week-old animals. Additionally, *db/db* mice consistently exhibited higher β-turn content compared to controls across all age groups. The ratio of 1689/1658, representing β-sheet content, also remained relatively constant, with a slight, but not statistically significant (P > 0.05), dip at 12 weeks followed by a notable increase at 24 weeks. As observed with β-turn content, *db/db* mice displayed higher β-sheet levels than controls across all groups.

Regarding secondary structures classified as aggregative, three band ratios were analysed. The 1612/1658 ratio, indicated a decreasing relative content of hydrated β-sheets with age. The difference between healthy and *db/db* mice also diminished over time, reaching statistical insignificance at 24 weeks. Interestingly, *db/db* mice exhibited higher hydrated β-sheet content at 7 weeks, lower at 12 weeks and no statistically significant difference (P > 0.05) at 24 weeks. Additionally, females tended to have lower hydrated β-sheet levels compared to males. The second aggregative ratio, 1639/1658, showed that the relative content of hydrated H-bonded β-turn remained nearly constant across all groups, except at 24 weeks, where *db/db* mice displayed significantly higher levels than controls. The 1700/1658 ratio, representing H-bonded antiparallel β-sheets, also remained stable except at 24 weeks, where a notable increase was observed in *db/db* mice. However, the relationship between control and *db/db* mice varied: *db/db* mice had higher values at 7 and 24 weeks but lower at 12 weeks, mirroring the trend seen for the 1612/1658 ratio.

Comparing results between whole RBCs and RBC membranes, some discrepancies were noted. A general agreement was observed for ratios 1639/1658, 1674/1658, 1689/1658, and 1700/1658, where overall trends in age and health conditions were consistent, although statistical significance varied. For 1612/1658, notable differences were found between whole RBCs and membranes. In RBC membranes, the relative content of hydrated β-sheets to α-helix slightly, but not significantly (P > 0.05), increased with age, with a significant rise at 24 weeks, and the increase in *db/db* mice was only evident at this time point. In contrast, in whole RBCs, the highest hydrated β-sheet content was observed in the youngest animals, decreasing with age. Differences between *db/db* and controls were most pronounced at 7 weeks (higher in *db/db*), slightly, but not significantly (P > 0.05), reversed at 12 weeks (higher in controls) and absent at 24 weeks.

The 1631/1658 ratio showed minor differences between whole RBCs and membranes. In whole RBCs, a slight, but not statistically significant (P > 0.05), increase in antiparallel β-sheets relative to α-helix was observed with age, with *db/db* mice showing lower values at 7 weeks but higher at 12 and 24 weeks. In RBC membranes, differences were only noticeable in the oldest animals, where *db/db* mice exhibited higher levels, but no consistent trends were detected.

## Conclusion

Our study presents an application of vibrational spectroscopy techniques to systematically characterize diabetes-induced biochemical alterations in RBC revealing their potential as biomarkers for disease progression. Through a comprehensive spectrochemical analysis, we identified significant structural and compositional changes in RBCs from diabetic (*db/db*) and control mice across different ages and sexes, providing new insights into diabetes-related erythrocyte dysfunction. Fig. 12 displays and summarizes the most important findings from all techniques.

**Fig. 12:**
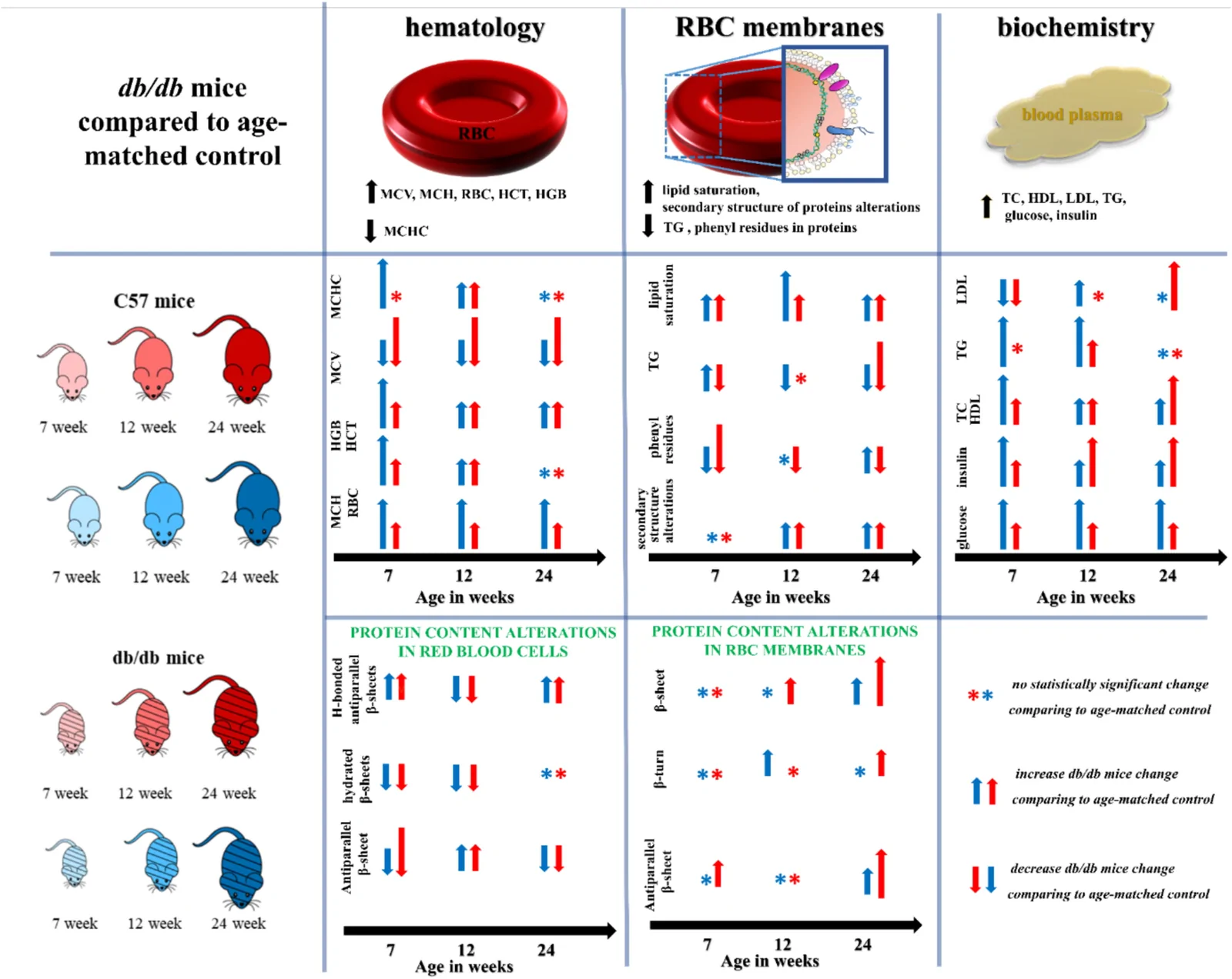
Schematic diagram illustrating changes in red blood cells and their membranes during aging, in relation to gender, and the progression of diabetes. (For interpretation of the references to colour in this figure legend, the reader is referred to the web version of this article.)

*Db/db* mice showed a significant increase in glucose levels that worsened with age, accompanied by pronounced obesity and insulin increase, more pronounced in females than males. Diabetic mice demonstrated increased MCV and MCH, along with decreased MCHC, consistent with haematopoietic dysregulation in the context of chronic hyperglycaemia and associated metabolic disturbances. Morphological changes in RBCs were linked to diabetic pathology, with sex-specific differences in MCH and MCHC for the oldest group. Elevated RBC count, HGB and HCT at 7 and 12 week old diabetic mice proves compensatory mechanism for hypoxia, without evidence of anemia. Dyslipidemia in *db/db* mice was marked by elevated plasma cholesterol, triglycerides (TG), HDL, and LDL levels, more pronounced in females, which exhibited higher weight gain and greater lipid accumulation, suggesting gender-specific differences in lipid metabolism.

For the first time, our study demonstrates the ability of FT-IR and RS to detect structural changes in RBC proteins with high specificity, distinguishing non-aggregative (α-helix, β-sheet, β-turn) from aggregative structures (hydrated β-sheet, H-bonded β-sheet, β-turns). The observed trends in protein aggregation, β-turn dynamics, and disulfide bond formation suggest a potential role of RBC protein alterations as novel biomarkers for diabetes progression and aging. Protein alterations are known to occur as part of the natural aging process [[70], [71], [72]]. However, the observed changes in erythrocyte membrane protein composition in *db/db* mice suggest disease-driven alterations. The most significant secondary structure changes were observed in 24-week-old animals, as confirmed with the oPLS-DA, suggesting that prolonged cellular stress accelerates protein misfolding. RBC membrane proteins exhibited reduced α-helix content and increased β-sheet accumulation, correlating with aggregation-prone conformations seen in diabetic pathology [10,11,73,74]. These membrane-specific alterations were more pronounced than in whole RBCs, underscoring the role of protein lipid interactions and prolonged exposure to the diabetic metabolic milieu, including chronic hyperglycaemia, in diabetes-related RBC dysfunction. Structural changes in diabetic females were more extensive, particularly in aggregative β-sheet structures. Modifications associated with chronic hyperglycaemia are linked to advanced glycation end products (AGEs), with sex-specific differences in lysine residue glycation suggesting a greater susceptibility in diabetic females [73,[75], [76], [77], [78]]. Diabetic RBCs exhibited increased β-sheet aggregation, particularly H-bonded antiparallel β-sheet and hydrated β-sheet structures, which contribute to membrane rigidity and dysfunction [[79], [80], [81]]. The role of β-turns in protein aggregation appears dual, potentially stabilizing proteins under stress but also promoting pathological aggregation [[82], [83], [84], [85], [86]]. Disulfide bonds, crucial for protein homeostasis and aggregation control, were significantly elevated in older diabetic mice, particularly in females. This increase likely reflects an adaptive response to oxidative stress, a hallmark of diabetes-induced metabolic disruption [[87], [88], [89], [90]]. In control mice, the amount of aggregated structures remained relatively stable between 7 and 24 weeks, with only males showing an increase in H-bonded antiparallel β-sheet content. In contrast, in *db/db* mice, significant structural changes were evident in females between 12 and 24 weeks, with increased β-sheet, hydrated β-sheet, H-bonded antiparallel β-sheet and H-bonded β-turn content relative to α-helix. Diabetes accelerates RBC membrane protein aggregation, predominantly in females. In contrast, control mice exhibited stable protein structures over time, with only males showing mild age-related aggregation [[70], [71], [72]]. An important conceptual point concerns the interpretation of the aromatic side-chain–associated Raman band at 1610 cm^−1^ [91]. In a complex matrix such as the RBC membrane, vibrational bands arise from the superposition of many protein species and are strongly modulated by their molecular environment. Therefore, the observed changes in the 1610/1447 ratio should not be interpreted as direct evidence for alterations in the absolute phenylalanine content of individual proteins. Instead, we consider this band a composite spectral marker that is sensitive to both the relative abundance of proteins enriched in aromatic residues and to matrix-dependent effects such as packing, hydration, oxidative modifications and lipid–protein interactions. Within this framework, our findings indicate robust, diabetes-related shifts in aromatic-side-chain–associated spectral features, but they do not specify which proteins are responsible. Future studies combining vibrational spectroscopy with quantitative proteomics of RBC membranes will be essential to identify the protein species underlying these signatures and to validate the mechanistic basis of the observed spectral changes [92].

Additionally, an analysis of the lipidic profile of RBC, along with blood plasma and body weight data, indicates sex-specific differences in lipid metabolism. In both *db/db* and healthy mice, TG levels increased with age in RBC membranes, with females showing higher levels across all groups. However, old *db/db* males exhibited a reduction in TG levels compared to controls. The fluctuation of TG levels in blood plasma, coupled with decreased TG in RBC membranes of diabetic animals, reflects a dysregulated lipid metabolism in diabetes. Triglycerides are critical for maintaining membrane lipid composition. The relationship between plasma lipids and RBC membrane lipids suggests that lipid distribution is altered in diabetic conditions. Elevated TGs in plasma, paired with reduced TG incorporation into RBC membranes, point to impaired lipid transport and utilization. This imbalance could reflect a broader dysregulation of lipid homeostasis in diabetes, with consequences for RBC function and membrane integrity. Additionally, the study shows a pronounced decrease in lipid unsaturation with age, more significant in diabetic animals. This trend towards higher saturated lipids in RBC membranes suggests reduced membrane fluidity, potentially impacting cellular flexibility and function in diabetes [93]. The findings indicate that diabetes-related alterations in RBCs may be associated with impaired RBC–endothelium interactions; while direct endothelial effects were not assessed, the observed RBC changes are consistent with processes known to accompany endothelial dysfunction and warrant further investigation.

Importantly, both FT-IR and Raman spectroscopy revealed statistically significant molecular alterations in the lipid and protein fractions of RBCs, demonstrating their complementary value in capturing the biochemical complexity of diabetes-related erythrocyte remodelling. While Raman spectroscopy successfully detected changes—particularly in the C–H stretching region—its diagnostic performance was limited compared to FT-IR. In contrast, FT-IR spectroscopy, especially within the protein amide regions, consistently outperformed Raman in statistical robustness, classification accuracy and biological interpretability. These results position the protein-associated FT-IR spectral window as the most diagnostically informative domain, offering the greatest potential for rapid, non-invasive staging of diabetes progression and sex-specific phenotyping through vibrational markers. PLS-DA applied to FT-IR data reliably discriminated between disease stages, sexes and age groups, emphasizing the technique’s specificity and interpretability. This targeted, spectrochemical insight provides a foundation for developing smart, minimally invasive diagnostic tools capable of capturing subtle, sex- and age-dependent molecular changes in erythrocyte structure—positioning FT-IR-based staging as a promising strategy in metabolic disease monitoring and precision medicine.

In line with the overarching vision of next-generation diabetes diagnostics, this study establishes a spectrochemical framework for staging diabetes-related alterations in RBCs within the db/db model, explicitly accounting for the combined effects of sex, age and a uniquely large experimental cohort (N = 120). Because our design compares untreated db/db mice with age-matched controls, the spectrochemical differences we report should be interpreted as robust associations between the diabetic metabolic milieu (including poor glycaemic control) and RBC remodelling, rather than being ascribed solely to hyperglycaemia. This multidimensional dataset provides a solid foundation for subsequent work aimed at validating the identified vibrational markers in human diabetic populations, thereby assessing their broader translational applicability. While the adoption of vibrational spectroscopy in routine clinical practice may require further advances in standardization, instrumentation and data analytics, ongoing developments including portable platforms and artificial intelligence–enabled analytical pipelines are rapidly increasing its feasibility. The translational promise of this approach is further supported by our previous studies demonstrating the successful use of both conventional Raman spectroscopy and SORS for non-invasive quality assessment of packed red blood cells using spectroscopic markers [[94], [95], [96]].

## Compliance with Ethics Requirement

All experiments were performed according to the Polish legislation, the ACT of January 15, 2015, on the protection of animals used for scientific or educational purposes and to the Directive 2010/63/EU (Chapter I, Article 3, Clause 1) of the European Parliament and of the Council. According to the latter, no additional approval is necessary when the animals sacrificed for tissue and blood collection are not subjected to any procedures beforehand. In this work, animals were not subject to any experimental procedures and only animal organs and tissues were used for analyses. In agreement with the Local Ethics Committee at the Institute of Immunology and Experimental Therapy, Polish Academy of Sciences in Wroclaw, an ethical review by was not required. All animal work was conducted in compliance with the ARRIVE guidelines.

## Declaration of competing interest

The authors declare that they have no known competing financial interests or personal relationships that could have appeared to influence the work reported in this paper.
