# Supplementary material for: Next-generation diabetes diagnostics: spectrochemical staging of red blood cells using vibrational spectroscopy
Source: J Adv Res. 2026 Jan 19;88:415–36. doi: 10.1016/j.jare.2026.01.052 (PMC13669592; doi:10.1016/j.jare.2026.01.052)
Supplement: Supplementary file 1 [file mmc1.pdf]

SUPLEMENTARY MATERIALS

Next-Generation Diabetes Diagnostics: Spectrochemical Staging of Red Blood Cells Using Vibrational Spectroscopy

Aleksandra Wilk[a,1], Natalia Wilkosz[a,1,], Marzena Rugiel[a], Marta Saluga[a], Kamil Kawon[a], Tetiana Stepanenko[a,b,c], Katarzyna Bulat[a], Aleksandra Wajda[d,e], Michał Szuwarzynski[f], Anna Zajaczkowska[g], Anna Morgunowicz[g], Grzegorz Chodaczek[g], Víctor Navarro-Esteve[h], David Perez-Guaita[h], Joanna Chwiej[a], Katarzyna M. Marzec[a,e,i,*]*

[a]AGH University of Krakow, Faculty of Physics and Applied Computer Science, al. A. Mickiewicza 30, 30-059 Krakow, Poland
[b] Jagiellonian University, Doctoral School of Exact and Natural Sciences, Lojasiewicza 11, 30-348 Krakow, Poland
[c] Jagiellonian University, National Synchrotron Radiation Centre SOLARIS, Czerwone Maki 98 Str., 30-392 Krakow, Poland
[d] Jagiellonian University, Faculty of Chemistry, Gronostajowa 2, 30-387 Krakow, Poland
[e] Faculty of Pharmacy, Jagiellonian University Medical College, 9 Medyczna Street, Krakow, 30-688, Poland
[f]Academic Centre for Materials and Nanotechnology, AGH University of Krakow, Al. Mickiewicza 30, 30-059 Krakow, Poland
[g] Lukasiewicz Research Network - PORT Polish Center for Technology Development, Stablowicka 147, 54-066 Wroclaw, Poland
[h] Department of Analytical Chemistry, University of Valencia, Dr. Moliner 50, Burjassot, Spain
[i]EBEAM Centre, Nanotechnology Centre, CEET, VSB–Technical University of Ostrava, Ostrava 708 00, Czech Republic

*** Corresponding author.** ***E-mail address: kmarzec@agh.edu.pl,*** **(K.M. Marzec),** ***nwilkosz@agh.edu.pl*** **(N.Wilkosz).**
[1] These authors contributed equally to this work.

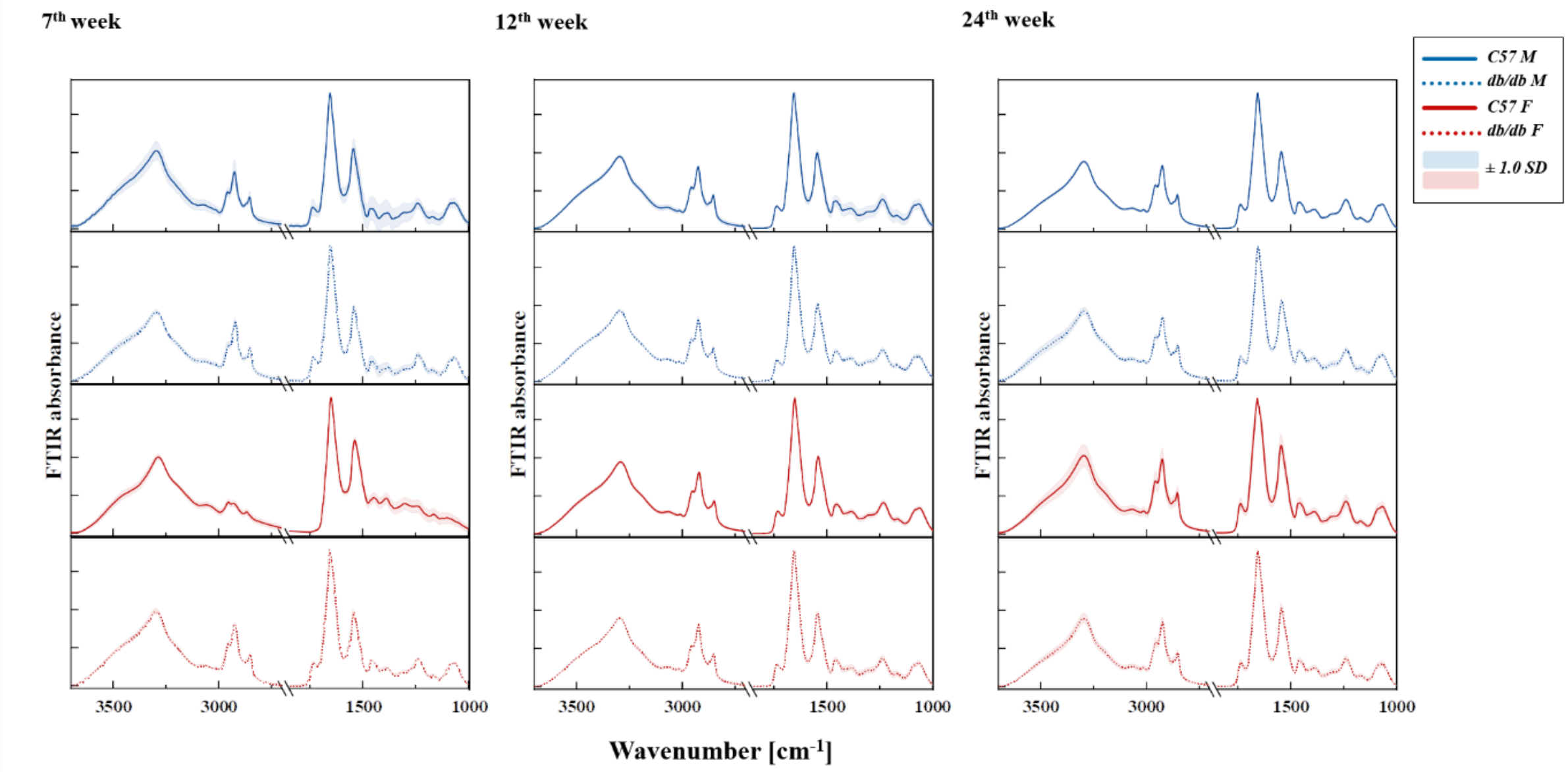

Supplementary Fig. S1. Average FT-IR spectra of RBC membranes from C57BL/6J (solid line) and db/db (dotted line) mice of both sexes (Male/M: blue, Female/F: red) at 7, 12, and 24 weeks of age across the 1000–3600 cm^{-1} range. The solid and dotted lines represent the group-averaged spectra, while the shaded area indicates the standard deviation.

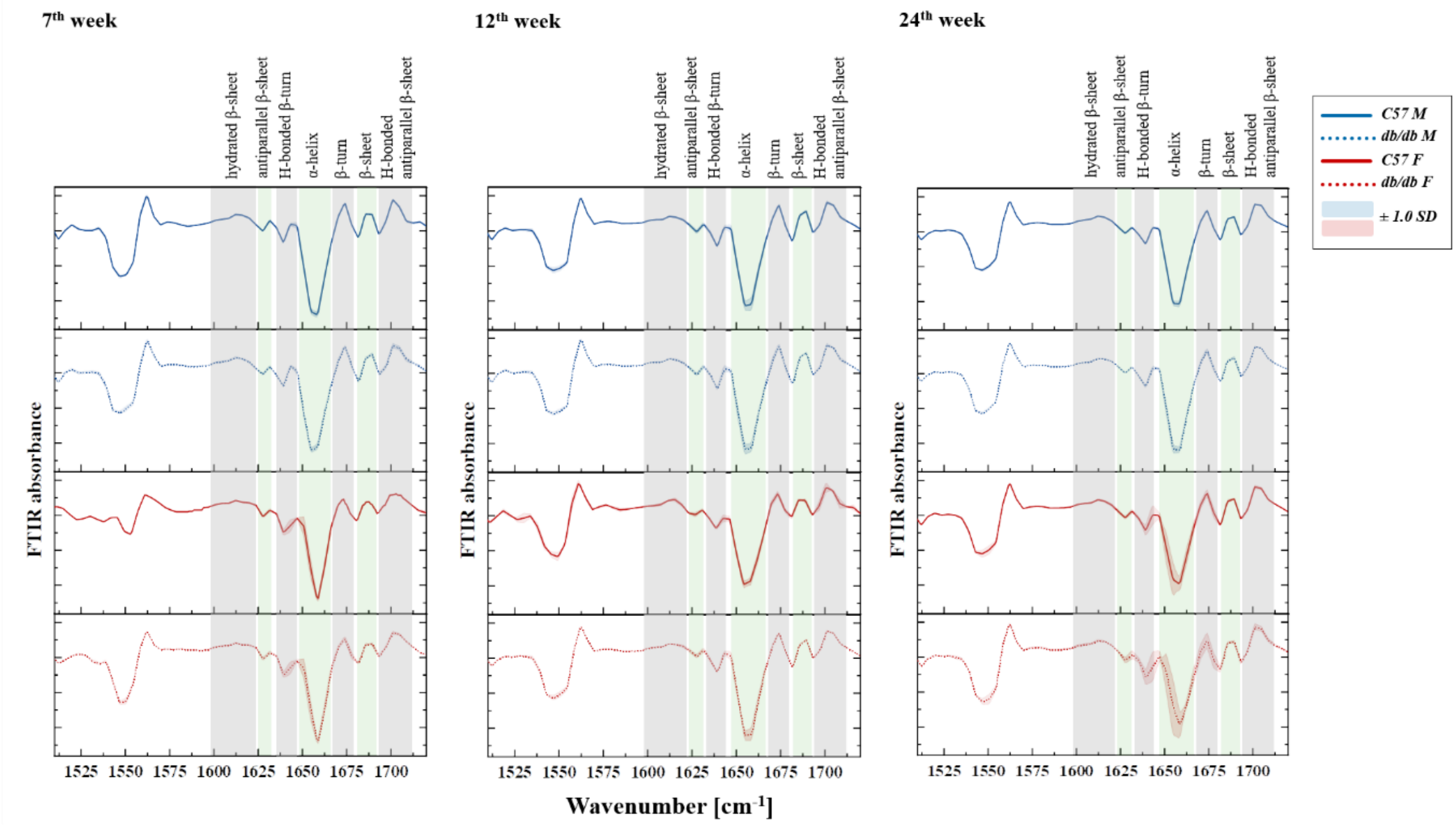

Supplementary Fig. S2. Second Derivatives of FT-IR Spectra for C57BL/6J (Solid Line) and db/db (Dotted Line) Mice of Both Sexes (Male/M: Blue, Female/F: Red) at 7, 12, and 24 Weeks of Age in the Protein-Associated Region (1510–1740 cm^{-1}). The lines represent the averaged second derivative spectra for each group, with shaded areas indicating the standard deviation. Grey shading marks the integration regions corresponding to specific analyzed bands, as detailed in the Figure.

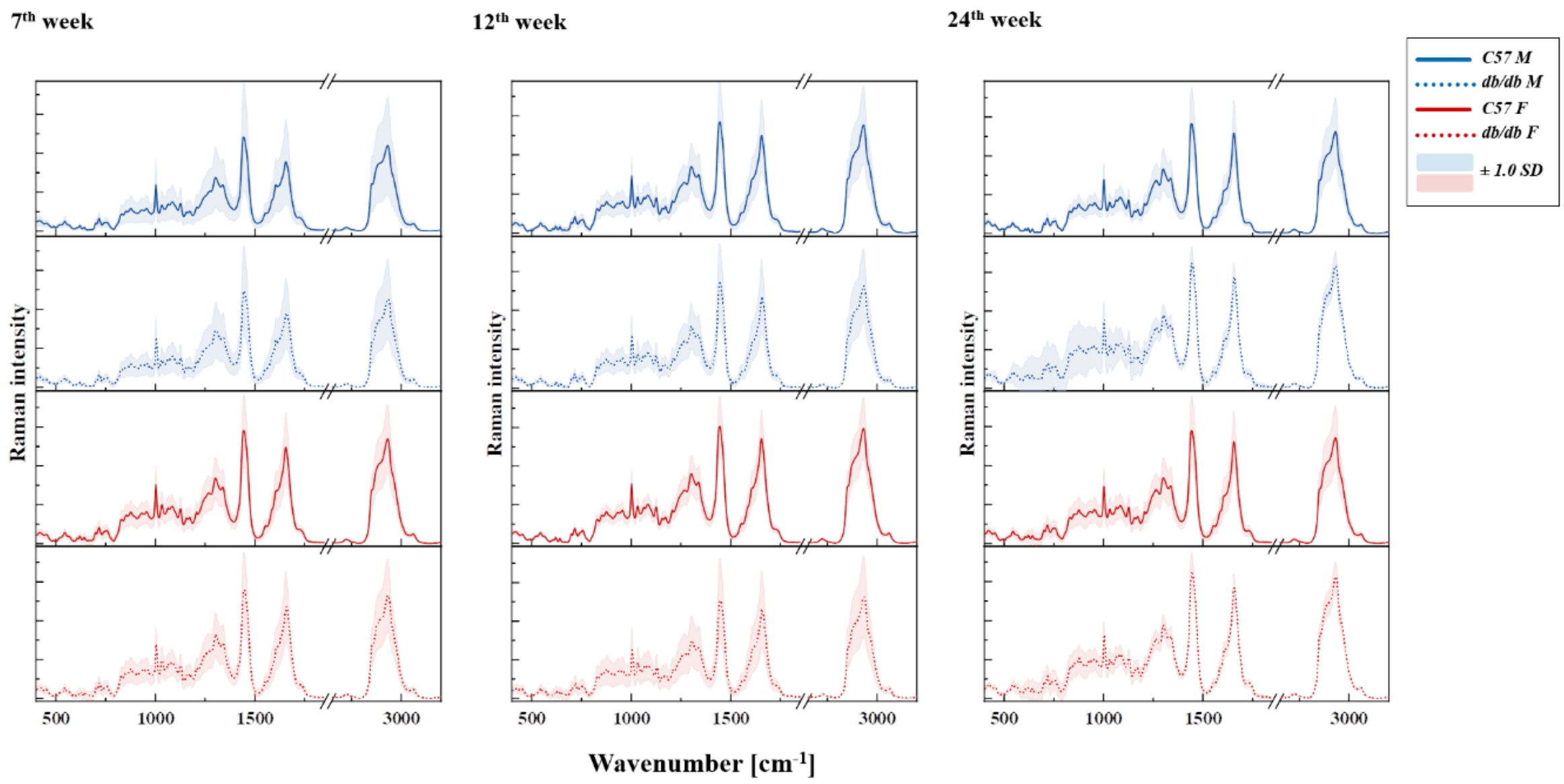

Supplementary Fig. S3. Average RS spectra of RBC membranes C57BL/6J (solid line) and db/db (dotted line) mice of both sexes (Male/M: Blue, Female/F: Red) at 7, 12, and 24 weeks of age across the 400–3200 cm⁻¹ range obtained with 532 nm excitation. The solid and dotted lines represent the group-averaged spectra, while the shaded area indicates the standard deviation.

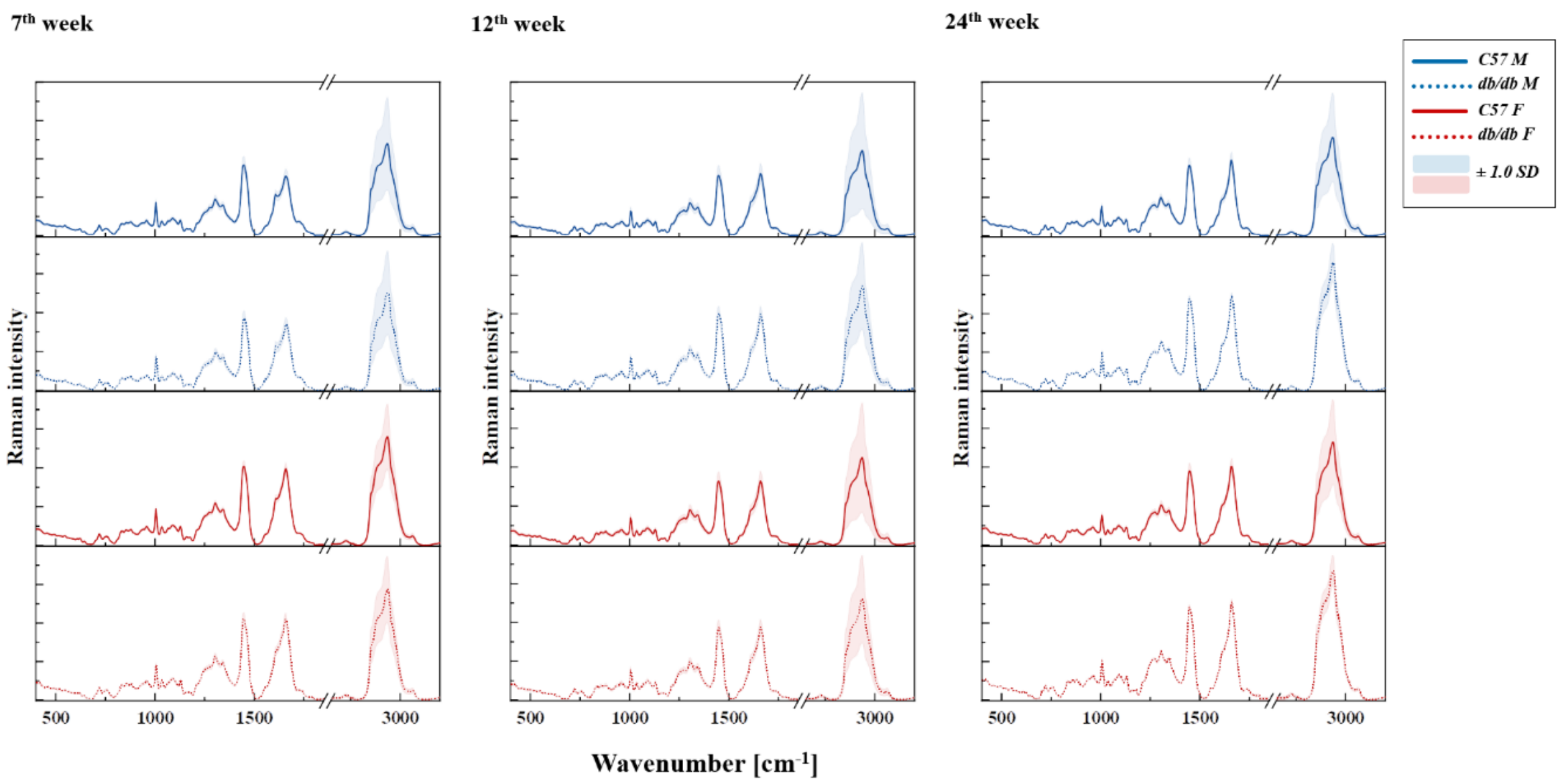

Supplementary Fig. S4. Average RS spectra of RBC membranes C57BL/6J (solid line) and db/db (dotted line) mice of both sexes (Male/M: Blue, Female/F: Red) at 7, 12, and 24 weeks of age across the 400–3200 cm⁻¹ range obtained with 488 nm excitation. The solid and dotted lines represent the group-averaged spectra, while the shaded area indicates the standard deviation.

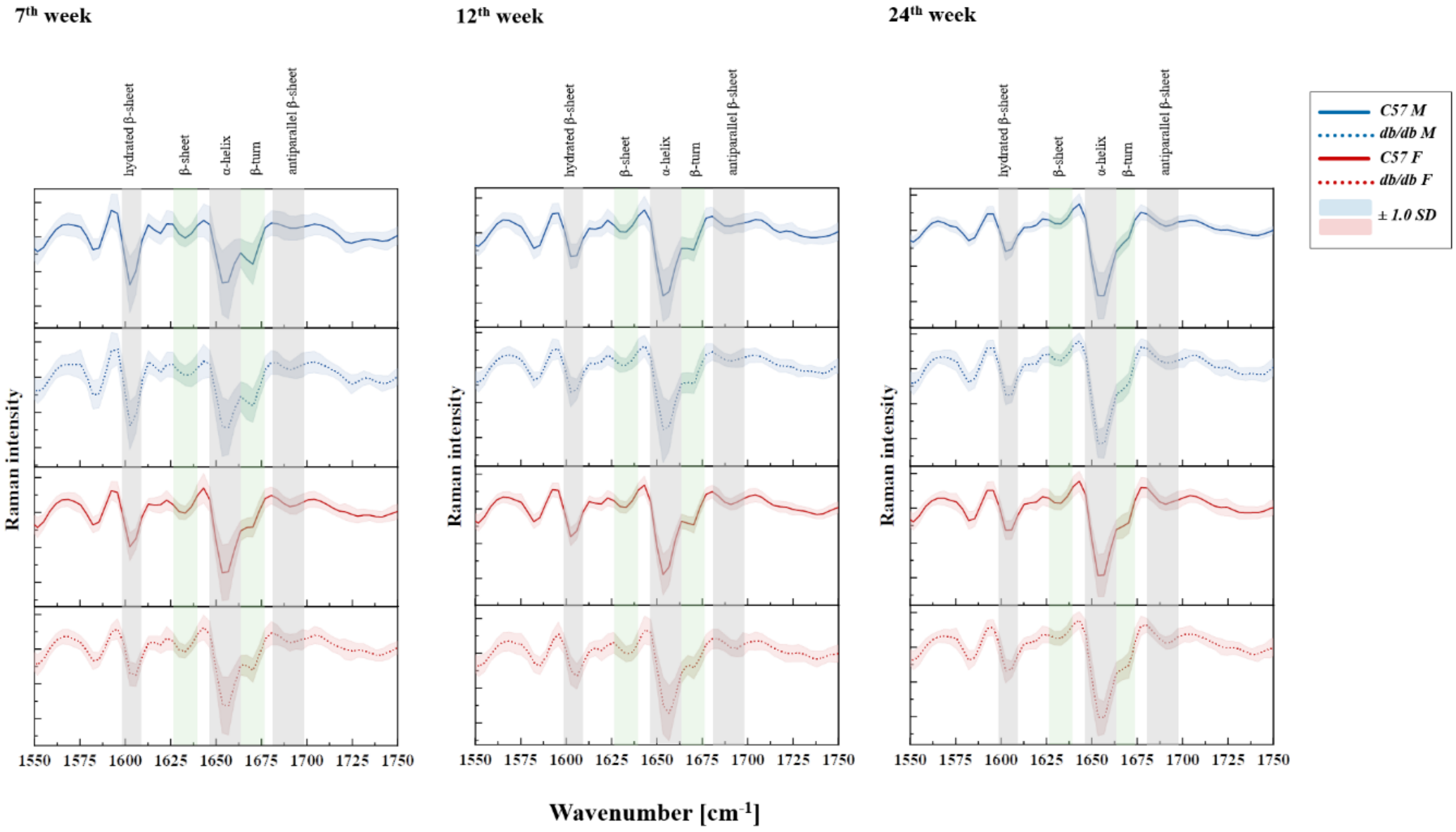

Supplementary Fig. S5. Second Derivatives of RS Spectra for C57BL/6J (Solid Line) and db/db (Dotted Line) Mice of Both Sexes (Male/M: Blue, Female/F: Red) at 7, 12, and 24 Weeks of Age in the Protein-Associated Region (1550–1750 cm⁻¹) obtained with 532 nm excitation. The lines represent the averaged second derivative spectra for each group, with shaded areas indicating the standard deviation. Grey shading marks the integration regions corresponding to specific analyzed bands, as detailed in the Figure.

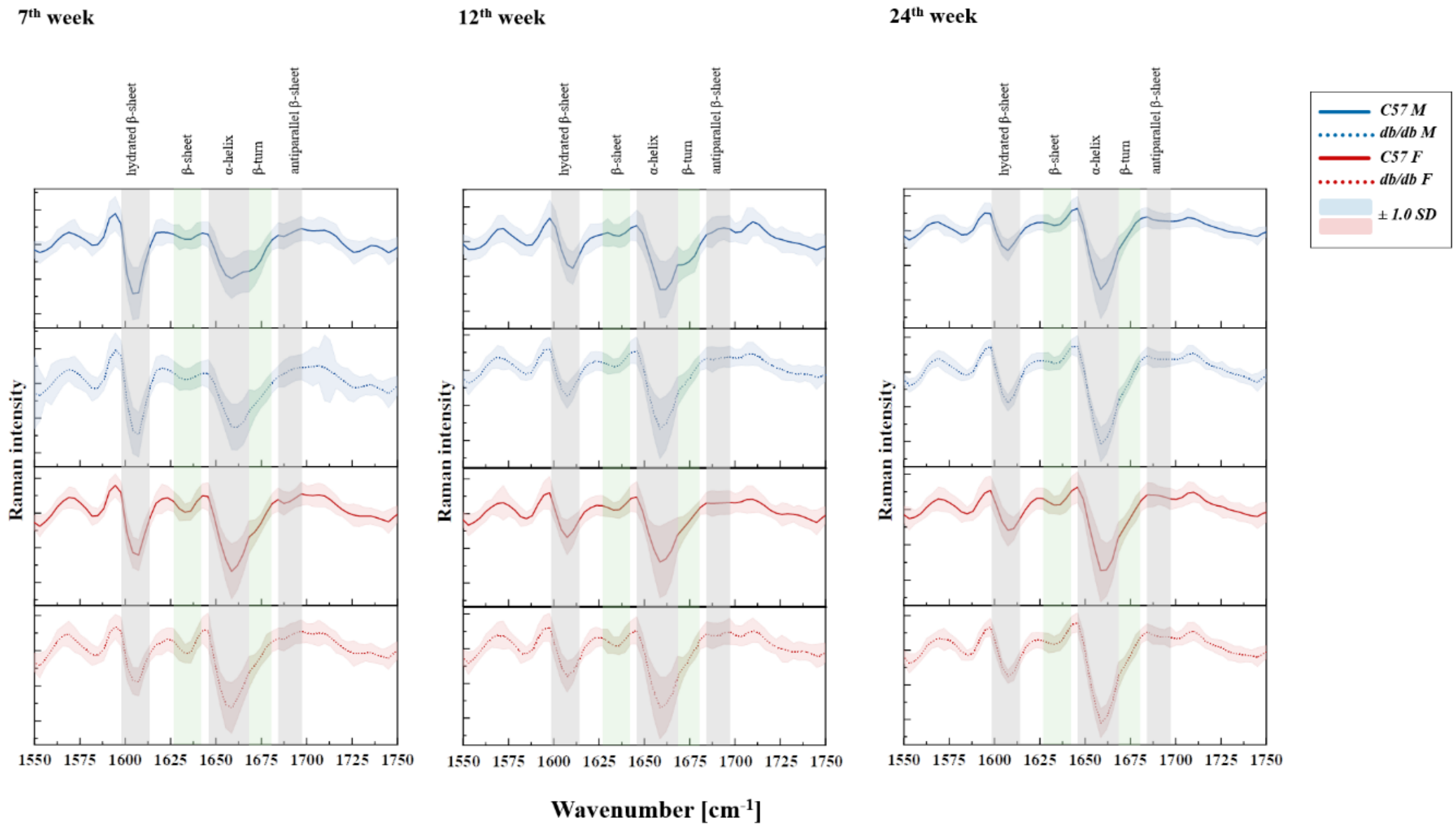

Supplementary Fig. S6. Second Derivatives of RS Spectra for C57BL/6J (Solid Line) and db/db (Dotted Line) Mice of Both Sexes (Male/M: Blue, Female/F: Red) at 7, 12, and 24 Weeks of Age in the Protein-Associated Region (1550–1750 cm⁻¹) obtained with 488 nm excitation. The lines represent the averaged second derivative spectra for each group, with shaded areas indicating the standard deviation. Grey shading marks the integration regions corresponding to specific analyzed bands, as detailed in the Figure.

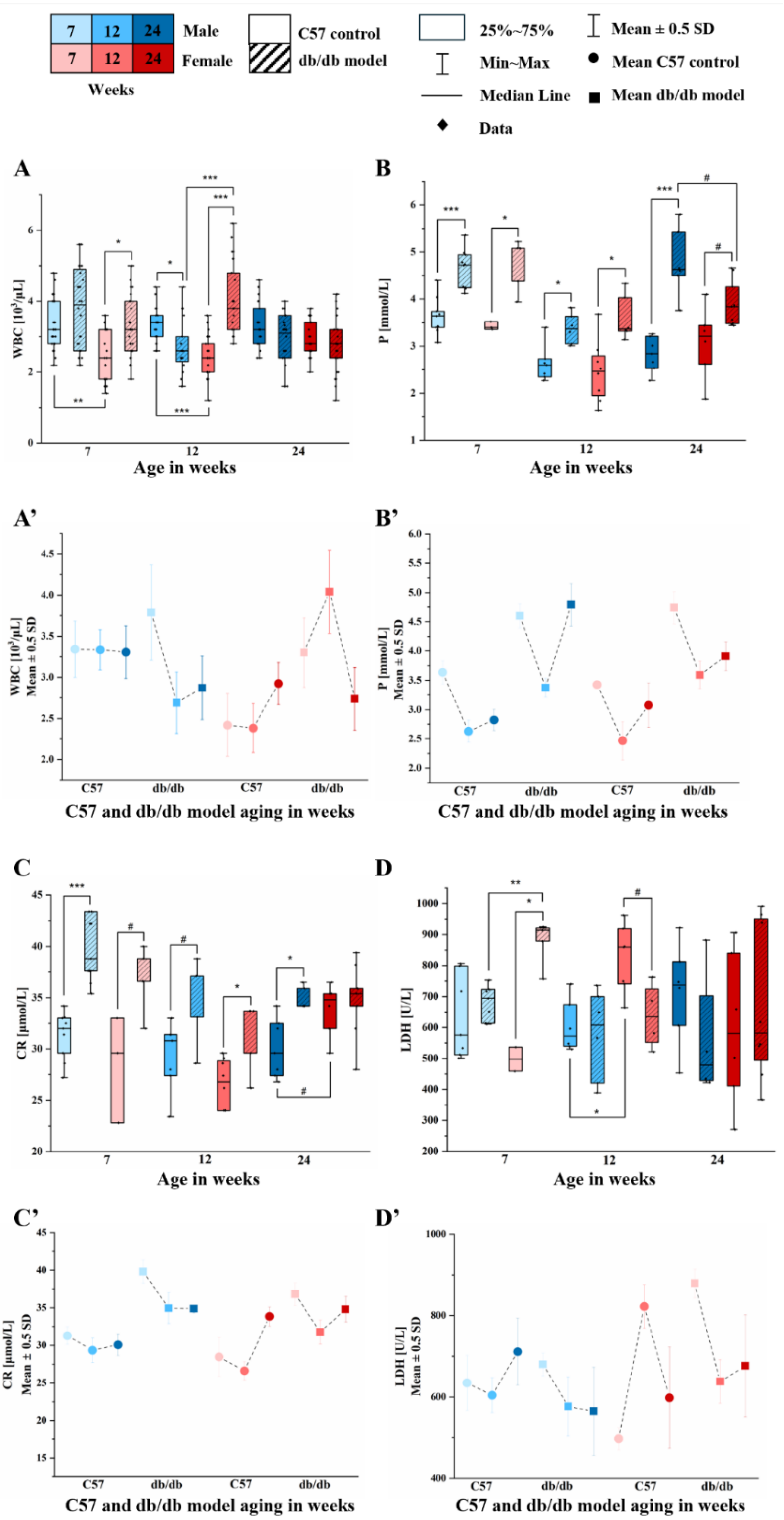

Supplementary Fig. S7. Changes in white blood cells (WBC) (A), phosphorus (P) (B), creatinine (CR) (C), and lactate dehydrogenase (LDH) (D) blood parameters over the progression of diabetes (at 7, 12, and 24 weeks of age) in male and female *db/db* mice compared to C57 controls. Data are presented as box plots (median with interquartile range and min-max whiskers). Normality was assessed using the Shapiro-Wilk test. Statistical significance was calculated using the Mann-Whitney test (#P < 0.05, *P < 0.01, **P < 0.001, ***P < 0.0001). Changes in mean values of white blood cells (WBC) (A'), phosphorus (P) (B'), creatinine (CR) (C'), and lactate dehydrogenase (LDH) (D') blood parameters over the progression of diabetes (at 7, 12, and 24 weeks of age) in male and female *db/db* mice compared to C57 controls. Data are presented as interval plots (mean with standard deviation whiskers).

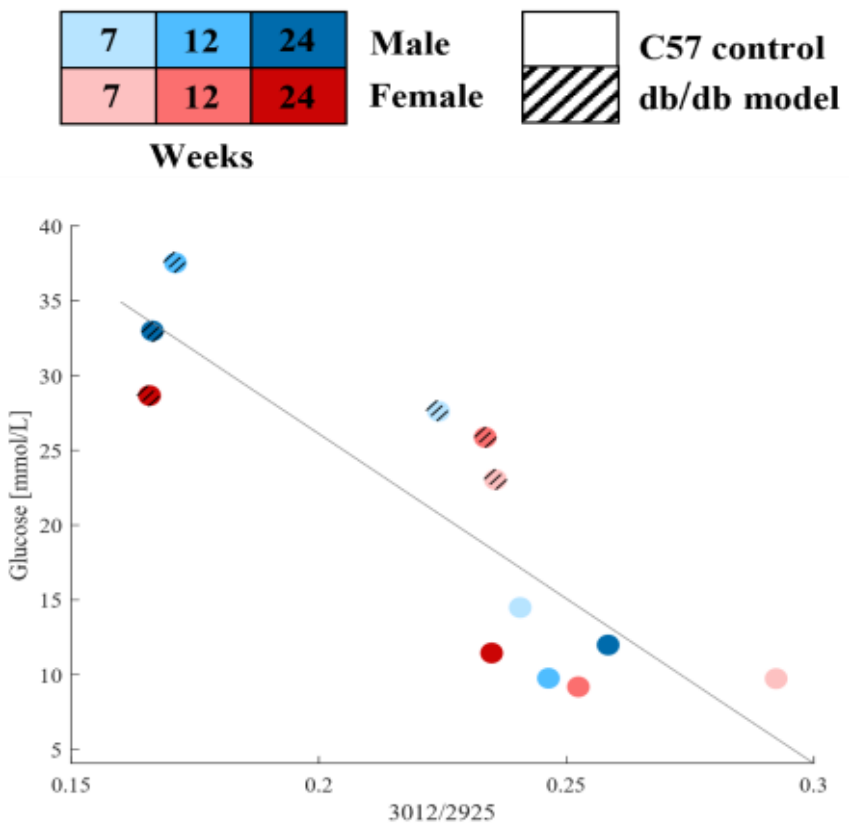

Supplementary Fig. S8. Correlation between the FTIR 3012/2925 lipid-unsaturation ratio of RBC membranes and plasma glucose levels. The plot includes a least-squares regression line and one point per group representing mean values.

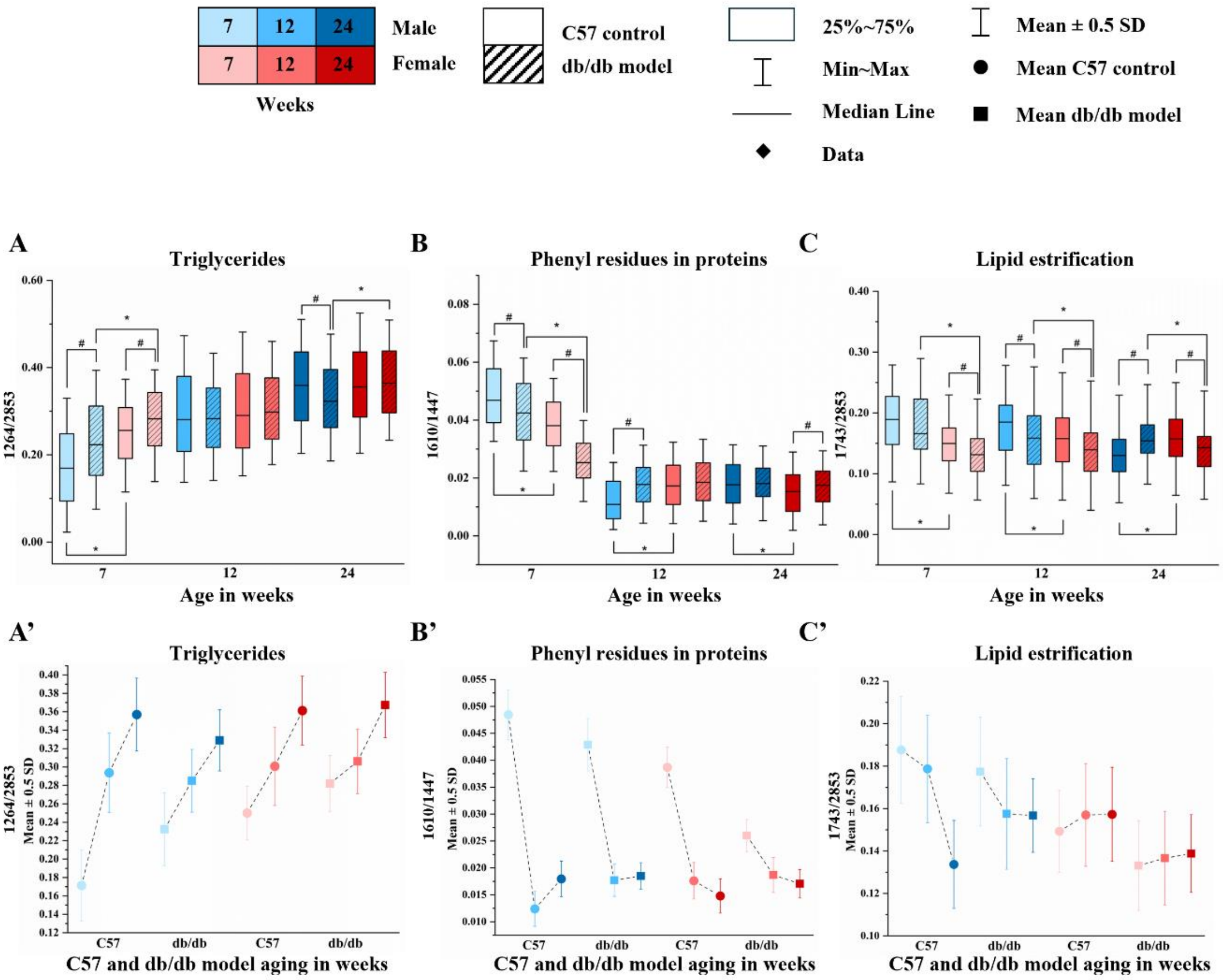

Supplementary Fig. S9. The ratios of the integral intensities of the selected (A-C) Raman bands (488 nm laser excitation) obtained from RBC membranes of 7, 12, and 24-week-old C57BL/6J and *db/db* mice. (A-C) The integration regions for the Raman bands include: scissoring and bending vibrations of CH_2 and CH_3 at 1743 cm^{-1} (1713-1762 cm^{-1}), symmetric CH_2 stretch at 2853 cm^{-1} (2841-2864 cm^{-1}), C–H bending at 1447 cm^{-1} (1422-1483 cm^{-1}), =C–H bending mode at 1264 cm^{-1} (1229-1279 cm^{-1}), and C=C stretch in aromatic rings at 1610 cm^{-1} (1587-1618 cm^{-1}). Normality was assessed using the Shapiro-Wilk test. Data are presented as box plots (median, min-max whiskers, and interquartile range), and significance was calculated using one-way ANOVA (#$P < 0.05$, *$P < 0.01$, **$P < 0.001$, ***$P < 0.0001$). Changes in mean Raman scattering (RS) ratios (A'-C') were calculated with the progression of diabetes (7, 12, and 24 weeks) in male and female *db/db* mice compared to age-matched C57 controls.

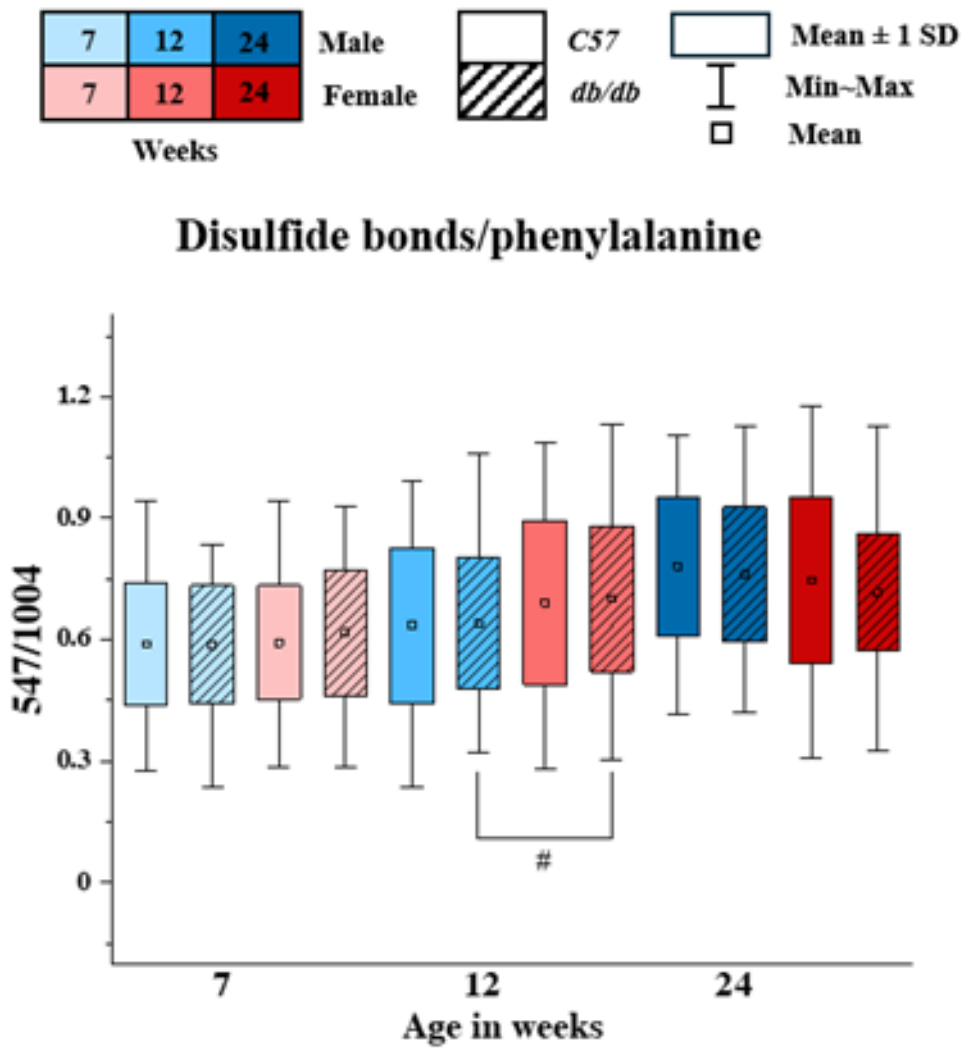

Supplementary Fig. S10. The ratio of integral intensities for RS bands (488 nm laser excitation) was analyzed to assess alterations in the tertiary structures of RBC membrane proteins for C57BL/6J and db/db Mice of Both Sexes (Male/M: Blue, Female/F: Red) at 7, 12, and 24 Weeks of Age. The 544/1004 cm^{-1} ratio corresponds to changes in disulfide bonds to phenylalanine. Normality was assessed using the Shapiro-Wilk test. Data are presented as box plots (mean, min–max whiskers, and standard deviation), and significance was calculated using one-way ANOVA (#P < 0.05, *P < 0.01, **P < 0.001, ***P < 0.0001).

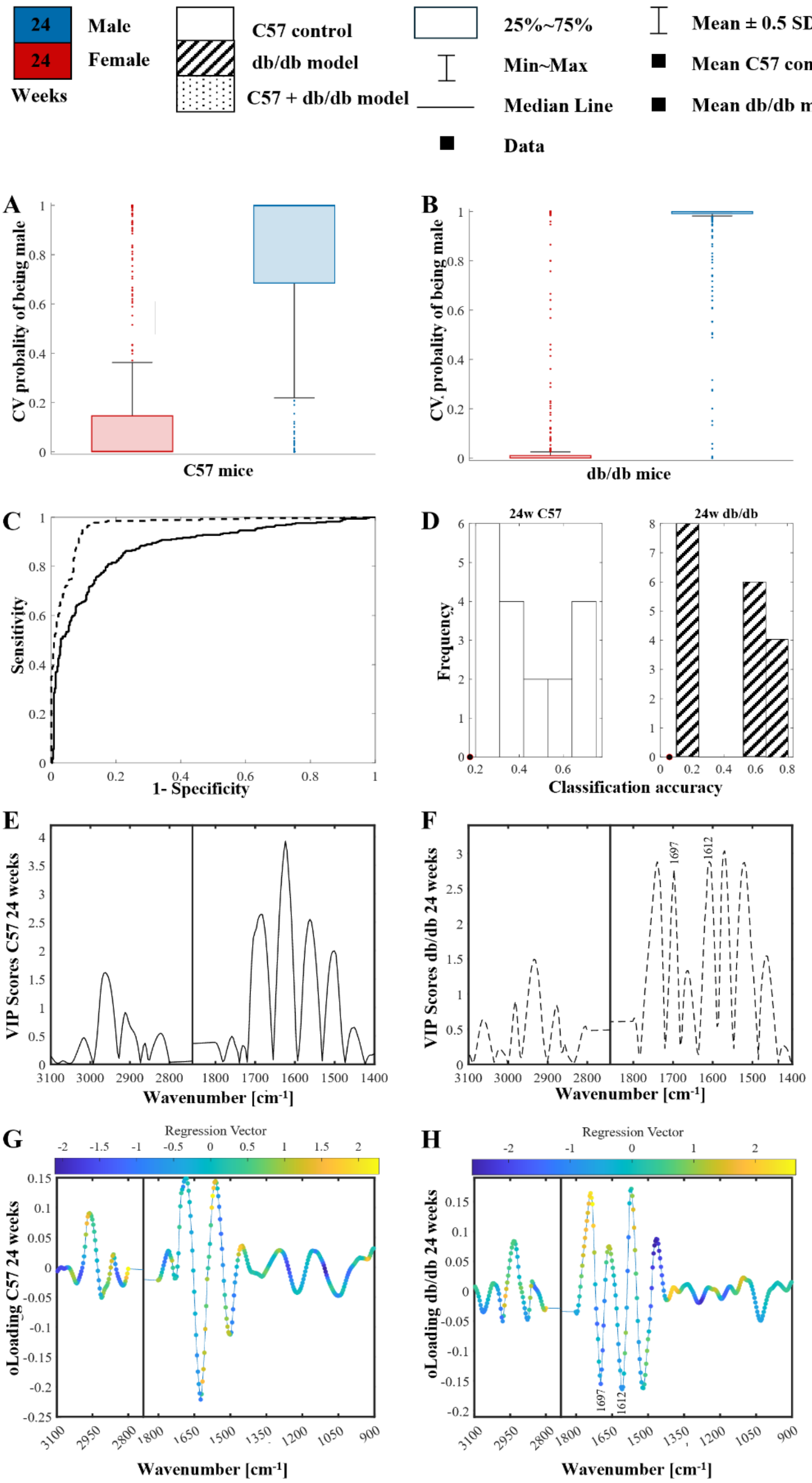

Supplementary Fig. S11. Boxplot of the CV probability of being classified as male of the PLSDA for control (A) and diabetic mice (B) according to FT-IR RBCs membranes. AUROC curve of the diabetic classification for C57 control and db/db model (dashed line) (C). Histogram of CV Errors of the classification of female vs. male obtained from permutation testing of the PLSDA model (D) for C57 control and db/db model (dashed). The black dot indicates the error of the actual, unpermuted data, which falls outside the main distribution of permuted values, highlighting the statistical significance P < 0.05) of the PLSDA model compared to random chance. VIP Scores of the PLSDA model for C57 control (E) and db/db mice (F). Orthogonalized loading coloured according to the regression vector for C57 control (G) and db/db mice (H).

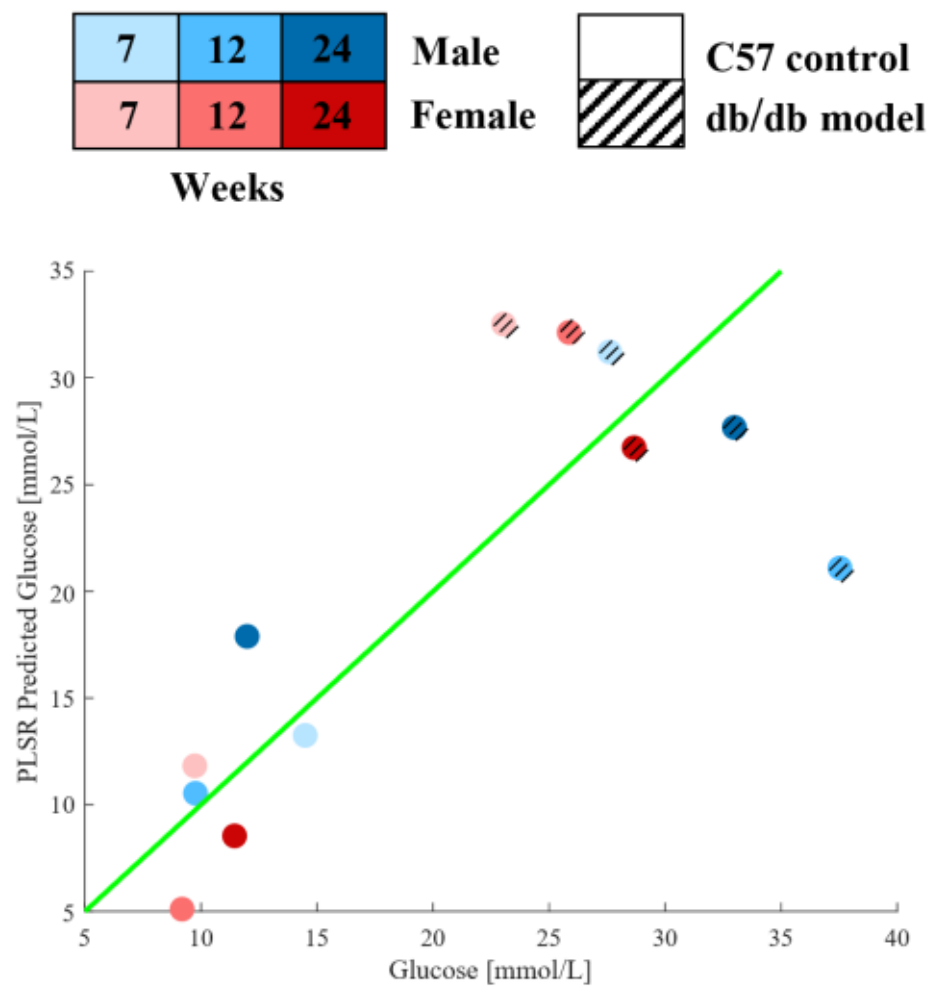

Supplementary Fig. S12. Comparison of measured plasma glucose levels with PLSR-predicted values obtained from RBC FTIR spectra. Each point represents the group mean, and the line indicates the 1:1 relationship between measured and predicted glucose. The model was built using the 1000–1400 and 2820–3100 cm^{-1} spectral regions and validated via leave-one-out cross-validation.

Supplementary Table 1. Comparison of estimated hematological and biochemical blood parameters in male and female *db/db* mice and C57 control mice at 7, 12 and 24 weeks of age, presented as mean ± standard deviation (SD), with the number (n) of measurements.

**7 week old**								
**Variable name**	**Males (Mean ± SD)**				**Females (Mean ± SD)**			
	**C57 control**	**n**	**db/db**	**n**	**C57 control**	**n**	**db/db**	**n**
**WBC [x10³/µL]**	3.34 ± 0.67	34	3.79 ± 1.14	28	2.42 ± 0.75	21	3.30 ± 0.83	30
**RBC [x10⁶/µL]**	8.77 ± 0.49	31	9.09 ± 0.42	31	8.43 ± 0.56	19	8.69 ± 0.57	26
**HGB [g/dL]**	14.10 ± 0.70	29	15.28 ± 0.64	29	13.69 ± 0.99	22	14.35 ± 0.76	26
**HCT [%]**	41.78 ± 3.04	36	46.16 ± 2.20	31	40.14 ± 3.46	23	43.08 ± 3.04	27
**MCV [fL]**	47.24 ± 0.81	34	50.67 ± 0.59	33	47.00 ± 0.29	24	49.74 ± 0.56	34
**MCH [pg]**	15.89 ± 0.21	30	16.84 ± 0.22	31	15.86 ± 0.25	18	16.21 ± 0.27	30
**MCHC [g/dL]**	33.64 ± 0.45	36	33.07 ± 0.50	35	33.82 ± 0.72	24	32.80 ± 0.57	34
**TC [mmol/L]**	2.65 ± 0.20	7	3.13 ± 0.24	11	2.06 ± 0.43	3	2.72 ± 0.16	5
**TG [mmol/L]**	1.76 ± 0.31	9	2.60 ± 0.45	9	1.97 ± 0.28	3	1.70 ± 0.14	4
**HDL [mmol/L]**	2.00 ± 0.12	7	2.19 ± 0.13	11	1.44 ± 0.28	3	2.00 ± 0.10	5
**LDL [mmol/L]**	0.49 ± 0.06	8	0.39 ± 0.03	10	0.48 ± 0.03	3	0.38 ± 0.04	4
**P [mmol/L]**	3.64 ± 0.37	9	4.60 ± 0.39	11	3.43 ± 0.07	3	4.74 ± 0.50	5
**CR [µmol/L]**	31.29 ± 2.20	9	39.82 ± 2.97	11	28.47 ± 4.24	3	36.80 ± 2.73	5
**LDH [U/L]**	635.01 ± 125.30	7	680.34 ± 51.63	7	497.80 ± 39.00	2	879.12 ± 63.39	5
**12 week old**								
**Variable name**	**Males (Mean ± SD)**				**Females (Mean ± SD)**			
	**C57 control**	**n**	**db/db**	**n**	**C57 control**	**n**	**db/db**	**n**
**WBC [x10³/µL]**	3.33 ± 0.48	34	2.69 ± 0.73	28	2.38 ± 0.59	21	4.04 ± 0.99	30
**RBC [x10⁶/µL]**	8.88 ± 0.62	31	9.57 ± 0.47	31	7.79 ± 0.73	19	9.51 ± 0.64	26
**HGB [g/dL]**	14.60 ± 1.33	19	15.77 ± 1.10	14	12.37 ± 1.21	19	16.00 ± 1.11	20
**HCT [%]**	40.72 ± 3.98	17	45.99 ± 3.64	15	35.86 ± 3.39	16	47.92 ± 4.03	22
**MCV [fL]**	46.52 ± 0.79	27	49.45 ± 1.32	20	46.15 ± 0.36	27	49.96 ± 0.84	27
**MCH [pg]**	16.20 ± 0.57	21	16.62 ± 0.27	17	16.02 ± 0.48	20	16.86 ± 0.33	22
**MCHC [g/dL]**	34.50 ± 1.18	18	33.52 ± 0.76	18	35.02 ± 1.17	22	33.99 ± 0.76	26
**TC [mmol/L]**	1.84 ± 0.41	7	2.72 ± 0.28	7	1.67 ± 0.22	8	2.79 ± 0.55	6
**TG [mmol/L]**	1.27 ± 0.44	7	3.31 ± 0.71	5	0.92 ± 0.10	7	2.07 ± 0.64	6

HDL [mmol/L]	1.40 ± 0.31	7	2.03 ± 0.15	7	1.24 ± 0.13	8	2.09 ± 0.35	6
LDL [mmol/L]	0.27 ± 0.05	7	0.40 ± 0.04	6	0.38 ± 0.03	8	0.38 ± 0.06	6
P [mmol/L]	2.63 ± 0.35	7	3.38 ± 0.29	6	2.47 ± 0.61	8	3.59 ± 0.43	6
CR [µmol/L]	29.34 ± 3.04	7	34.94 ± 3.68	5	26.61 ± 2.25	8	31.77 ± 2.90	6
LDH [U/L]	604.62 ± 77.74	6	576.88 ± 132.53	6	822.39 ± 99.32	7	638.13 ± 93.06	4
**24 week old**								
**Variable name**	**Males (Mean ± SD)**				**Females (Mean ± SD)**			
	**C57 control**	**n**	**db/db**	**n**	**C57 control**	**n**	**db/db**	**n**
WBC [x10^3/µL]	3.31 ± 0.62	34	2.87 ± 0.76	28	2.92 ± 0.50	21	2.74 ± 0.75	30
RBC [x10^6/µL]	8.58 ± 0.81	31	8.07 ± 0.79	31	8.90 ± 1.04	19	8.98 ± 0.78	26
HGB [g/dL]	12.86 ± 1.64	19	13.28 ± 1.31	18	14.31 ± 1.42	23	14.38 ± 1.02	24
HCT [%]	35.99 ± 4.49	17	38.96 ± 4.10	18	40.63 ± 4.80	20	41.92 ± 2.99	20
MCV [fL]	44.35 ± 0.62	26	48.83 ± 1.43	24	44.83 ± 1.11	24	48.96 ± 1.53	27
MCH [pg]	15.47 ± 0.47	23	16.95 ± 0.53	24	15.69 ± 0.45	19	16.37 ± 0.40	20
MCHC [g/dL]	35.13 ± 1.20	23	33.94 ± 0.55	17	34.03 ± 0.60	20	33.06 ± 0.87	25
TC [mmol/L]	2.11 ± 0.22	7	3.56 ± 0.78	6	1.69 ± 0.18	6	3.90 ± 0.52	8
TG [mmol/L]	1.45 ± 0.23	7	1.68 ± 0.15	4	1.23 ± 0.32	6	1.56 ± 0.38	8
HDL [mmol/L]	1.62 ± 0.14	7	2.69 ± 0.53	6	1.23 ± 0.15	6	3.04 ± 0.37	9
LDL [mmol/L]	0.34 ± 0.07	7	0.48 ± 0.16	6	0.38 ± 0.04	6	0.55 ± 0.06	6
P [mmol/L]	2.83 ± 0.34	7	4.79 ± 0.66	6	3.08 ± 0.69	6	3.91 ± 0.46	9
CR [µmol/L]	30.07 ± 2.65	7	34.87 ± 0.96	6	33.85 ± 2.36	6	34.80 ± 3.17	9
LDH [U/L]	711.45 ± 149.21	6	565.43 ± 186.93	4	598.35 ± 226.78	6	676.55 ± 233.98	8

Abbreviation: WBC: White Blood Cells; RBC: Red Blood Cells; HGB: Haemoglobin; HCT: Hematocrit; MCV: Mean Corpuscular Volume; MCH: Mean Corpuscular Haemoglobin; MCHC: Mean Corpuscular Haemoglobin Concentration; TC: Total Cholesterol; TG: Triglyceride; HDL: High-Density Lipoprotein; LDL: Low-Density Lipoprotein; P: Phosphorus; CR: Creatinine; LDH: Lactate Dehydrogenase.

Supplementary Table 2. Confusion matrices for the SVM-DA models for FT-IR whole RBCs

	**24 week old Females**		**24 week old Males**	
	**Actual class**		**Actual class**	
	**C57 control**	**db/db**	**C57 control**	**db/db**
**Predicted as C57 Control**	305	105	176	19
**Predicted as db/db**	0	106	43	89
